# Human T-bet governs the generation of a distinct subset of CD11c^high^CD21^low^ B cells

**DOI:** 10.1126/sciimmunol.abq3277

**Published:** 2022-07-22

**Authors:** Rui Yang, Danielle T. Avery, Katherine J. L. Jackson, Masato Ogishi, Ibtihal Benhsaien, Likun Du, Xiaofei Ye, Jing Han, Jérémie Rosain, Jessica N. Peel, Marie-Alexandra Alyanakian, Bénédicte Neven, Sarah Winter, Anne Puel, Bertrand Boisson, Kathryn J. Payne, Melanie Wong, Amanda J. Russell, Yoko Mizoguchi, Satoshi Okada, Gulbu Uzel, Christopher C. Goodnow, Sylvain Latour, Jalila El Bakkouri, Aziz Bousfiha, Kahn Preece, Paul E. Gray, Baerbel Keller, Klaus Warnatz, Stéphanie Boisson-Dupuis, Laurent Abel, Qiang Pan-Hammarström, Jacinta Bustamante, Cindy S. Ma, Jean-Laurent Casanova, Stuart G. Tangye

**Affiliations:** 1 St Giles Laboratory of Human Genetics of Infectious Diseases, Rockefeller Branch, Rockefeller University, New York, NY 10065, USA; 2 Department of Pediatrics, Weill Cornell Medicine, New York, NY, 10065, USA; 3 Garvan Institute of Medical Research, Darlinghurst 2010, NSW Australia; 4 Laboratory of Clinical Immunology, Inflammation, and Allergy, Faculty of Medicine and Pharmacy of Casablanca, King Hassan II University, 20460 Casablanca, Morocco; 5 Clinical Immunology Unit, Department of Pediatric Infectious Diseases, Children's Hospital, CHU Averroes, 20460 Casablanca, Morocco; 6 Department of Biosciences and Nutrition, Karolinska Institutet, 17177 Stockholm, Sweden, EU; 7 Laboratory of Human Genetics of Infectious Diseases, Necker Branch, INSERM UMR 1163, Necker Hospital for Sick Children, 75015 Paris, France; 8 Paris Cité University, Imagine Institute, 75015 Paris, France; 9 Immunology Laboratory, Necker Hospital for Sick Children, Assistance Publique-Hôpitaux de Paris (AP-HP), 75015 Paris, France, EU; 10 Department of Pediatric Immunology, Hematology and Rheumatology, Necker Hospital for Sick Children, AP-HP, Paris, France; 11 Laboratory of Lymphocyte Activation and Susceptibility to EBV Infection, INSERM UMR 1163, Imagine Institute, 75015 Paris, France.; 12 St Vincent’s Clinical School, Faculty of Medicine, UNSW Sydney, Darlinghurst 2010, Australia; 13 Children’s Hospital at Westmead, NSW, Australia; 14 Faculty of Medicine, University of Sydney, Sydney, NSW, Australia; 15 Department of Pediatrics, Hiroshima University, Graduate School of Biomedical and Health Sciences, Hiroshima, Japan.; 16 Laboratory of Clinical Immunology and Microbiology, National Institute of Allergy and Infectious Diseases, National Institutes of Health, Bethesda, MD.; 17 John Hunter Children's Hospital, Newcastle, New South Wales, Australia.; 18 School of Women's and Children's Health, UNSW Sydney, Sydney, New South Wales, Australia.; 19 Department of Immunology and Infectious Diseases, Sydney Children's Hospital, Sydney, New South Wales, Australia.; 20 Department of Rheumatology and Clinical Immunology, Medical Center - University of Freiburg, Faculty of Medicine, University of Freiburg, Freiburg, Germany.; 21 Center for Chronic Immunodeficiency (CCI), Medical Center - University of Freiburg, Faculty of Medicine, University of Freiburg, Freiburg, Germany; 22 Study Center for Primary Immunodeficiencies, Necker Hospital for Sick Children, AP-HP, 75015 Paris, France; 23 Howard Hughes Medical Institute, New York, NY, USA; 24 Department of Pediatrics, Necker Hospital for Sick Children, AP-HP, 75015 Paris, France

## Abstract

High level expression of the transcription factor T-bet characterizes a phenotypically distinct murine B-cell population known as ‘age-associated B cells’ (ABCs). T-bet-deficient mice have reduced ABCs and impaired humoral immunity. We describe a patient with inherited T-bet deficiency and largely normal humoral immunity including intact somatic hypermutation, affinity maturation and memory B-cell formation *in vivo*, and B-cell differentiation into Ig-producing plasmablasts *in vitro*. Nevertheless, the patient exhibited skewed class switching to IgG1, IgG4 and IgE, along with reduced IgG2, both *in vivo* and *in vitro*. Moreover, T-bet was required for the *in vivo* and *in vitro* development of a distinct subset of human B cells characterized by reduced expression of CD21, and the concomitantly high expression of CD19, CD20, CD11c, FCRL5, and T-bet, a phenotype which shares many features with murine ABCs. Mechanistically, human T-bet governed CD21^lo^CD11c^hi^ B cell differentiation by controlling chromatin accessibility of lineage-defining genes in these cells: *FAS*, *IL21R*, *SEC61B*, *DUSP4*, *DAPP1*, *SOX5*, *CD79B* and *CXCR4*. Thus, human T-bet is largely redundant for long-lived protective humoral immunity but is essential for the development of a distinct subset of human CD11c^hi^ CD21lo B cells.

## One sentence summary

Germline mutation of *TBX21* reveals a requirement for T-bet in the generation of a distinct subset of CD11c^hi^CD21^lo^ human B cells.

## Introduction

T-bet was originally discovered as a T helper 1 (T_H_1) cell-specific lineage-determining transcription factor in mice (1, 2). Its pleiotropic role in innate and adaptive immunity has been extended to include regulation of the development and function of murine dendritic cells (DCs), NK, NKT, B, and γδ T cells (3). T-bet is expressed by murine NK and T cells (3), as well as a small subset of murine B cells phenotypically defined as CD23^−^CD21/35^lo^ or CD11c^+^CD11b^+^. These cells have been referred to as “age-associated” B cells (ABCs), as their frequency increases in lymphoid tissues of aging mice (4-9). T-bet expression is induced in murine B cells in response to activation via Toll-like receptors (TLR4, 7, 9), CD40, and cytokines including IFN-γ, IL-12, IL-18, IL-21, and IL-27 (1, 7, 8, 10-14). Studies of *Tbx21*^*−/−*^ mice showed that T-bet functions intrinsically in B cells to modulate class-switch recombination (CSR) to and production of IgG subclasses, including IgG2a/c (10, 11, 15-17). A non-redundant role for T-bet in IgG2a/c CSR has been validated in mouse models of viral and parasitic infections (18-22) and NP-KLH immunization (16). B cell-intrinsic T-bet can also orchestrate IFN-γ-induced formation of long-lived antibody-secreting cells in mouse models of viral infection (17). Together, these studies revealed a key B-cell intrinsic role for T-bet in regulating the quality of murine humoral immune responses.

T-bet has also been implicated in fate decisions for murine B-cell subsets. ABC, as a subset of T-bet^+^ B cells, has been the focus of growing interest over the last decade (4, 6, 9, 23). In mice, numbers of these cells increase in lymphoid tissues with age (4, 23, 24), as well as following infection with certain viruses (6, 25), bacteria (26, 27), parasites (28), and in the context of autoimmunity (29-31). Depletion of ABCs in mouse models of systemic lupus erythematosus (SLE) decreases anti-chromatin IgG levels (32) and disease severity (30), but the non-redundant function of ABCs remains much less well understood than the conditions in which this B-cell subset expands in mice. Moreover, T-bet expression in murine B cell subsets has yet to be clearly defined, as it is detected in only ~50% of all ABCs (8, 9). It is unclear whether this heterogeneity results from the presence of distinct subsets of ABCs, or simply the existence of numerous differentiation stages of the same ABC lineage. Furthermore, the requirement for T-bet in the development of murine ABCs *in vivo* is a matter of debate, as some studies indicated T-bet is indispensable for ABC formation (6, 30), whereas others showed ABCs are generated normally from T-bet deficient B cells (33, 34). It is thus unclear if T-bet is essential or redundant for generating ABCs or a subset of ABCs.

In humans, numerous subsets of B cells resembling murine ABCs have been identified. These include CD21^lo^T-bet^+^ B cells that were first discovered to be increased in some individuals with common variable immunodeficiency (CVID) (35). Subsequent studies identified similar populations that have been termed “activated naïve-like B cells”, “atypical memory”, or “IgD^−^CD27^−^ double-negative” B cells that are significantly expanded in as chronic viral (HIV, HCV) and malaria infections (36-42), and autoimmune conditions including SLE and rheumatoid arthritis (9, 31, 43-49). CD21^lo^T-bet^+^ B cells are also detected at increased frequencies following influenza vaccination (50, 51) and SARS-CoV-2 infection causing severe COVID19 (52, 53). Thus, depending on the condition, human CD21^lo^T-bet^+^ B cells might be pathogenic, protective, or both. In contrast to mice, CD21^lo^T-bet^+^ B cells only mildly increase until 30 years of age but do not increase afterwards (54). Moreover, the role of T-bet in the development, maintenance, and function of human B cells, including CD21^lo^T-bet^+^ B cells, and humoral immunity in response to pathogen infections, is completely unknown.

We recently reported the first patient with autosomal recessive (AR) complete T-bet deficiency (55, 56). This patient carries a homozygous in-del mutation in *TBX21* that abolishes DNA-binding activity without abrogating protein expression (55). By disrupting the development of IFN-γ-producing innate or innate-like adaptive lymphocytes, human T-bet deficiency causes Mendelian susceptibility to mycobacterial disease (55). The patient also developed upper airway inflammation and peripheral eosinophilia due to T_H_2-skewing of T-bet-deficient CD4^+^ T cells (56). However, the development and function of B cells in this patient have not been studied. Human T-bet deficiency therefore provides a unique opportunity to determine the physiological requirement for T-bet in the induction and maintenance of humoral immunity, and the development and homeostasis of T-bet-expressing B cells. By thoroughly assessing B cells in inherited T-bet deficiency, we delineated the essential and redundant roles of human T-bet in humoral immunity and serological memory.

## Results

### Altered class switch recombination and production of polyclonal antibodies in human T-bet deficiency

**Figure 1.**
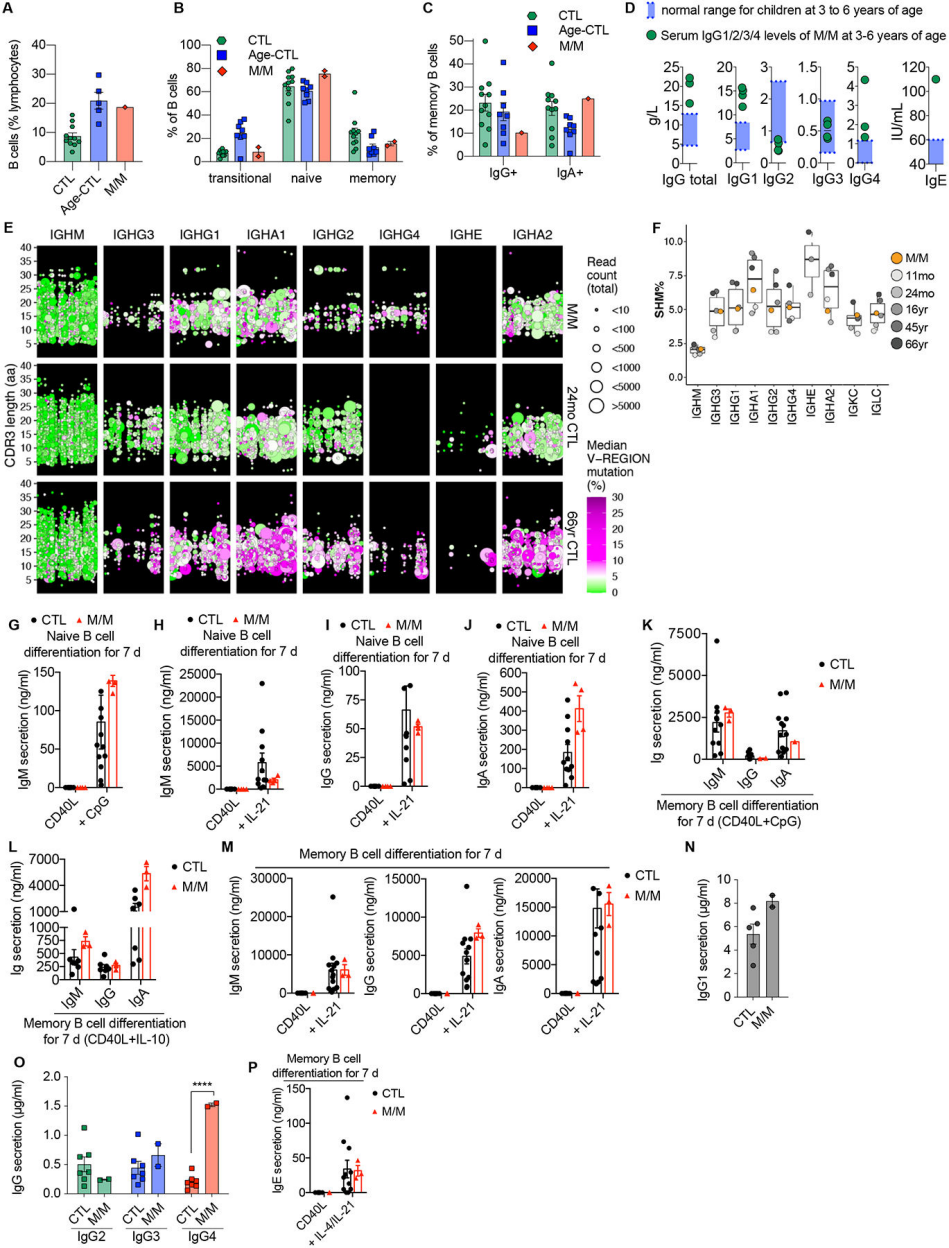
*In vivo* development and *in vitro* function of B cells from a patient with inherited human T-bet deficiency. **(A)** Percentages of B cells among live lymphocytes gated from PBMCs of healthy donors (CTL), age-matched healthy donors (Age-CTL) or a patient (P or M/M) with inherited complete T-bet deficiency. **(B)** Percentages of naïve, memory and transitional B-cell subsets among B cells as in (A). **(C)** Percentages of IgG^+^ or IgA^+^ B cells among the memory B cells of CTL, age-matched CTL or P, measured by conventional flow cytometry (FACS). **(D)** Levels of total IgG, IgG1, IgG2, IgG3, IgG4, and IgE in plasma samples from P (M/M) and their normal range in the age-matched group (3 – 6 years of age). **(E)** Overview of the IGH repertoire for P (top row) and control donors aged 24 months (middle row) and 66 years (bottom row). Each column displays clones from a different isotype subclass. Each point is a B-cell clone, with point size scaled for clone size and colored according to the clone’s median SHM rate. Clones are positioned on the basis of V usage (*x*-axis) and CDR3 length (*y*-axis) with some jitter to prevent overplotting. **(F)** Points show the donor mean or median SHM for each IGH isotype, IGK and IGL repertoires; box plots summarize the median and interquartile ranges for all 6 donors. **(G)** Naïve B cells, isolated by FACS, from CTL or P were stimulated with CD40 ligand (CD40L) in the presence or absence of CpG_2006_ oligodeoxynucleotides (CpG) for 7 days. IgM levels in culture supernatants were determined by ELISA. **(H - J)** Naive B cells, as in (G), were stimulated with CD40L in the presence or absence of IL-21 for 7 days. Levels of IgM (H), IgG (I) or IgA (J) in culture supernatants were determined by ELISA. **(K - M)** Memory B cells, isolated by FACS, were stimulated with CD40L in the presence of CpG (K), IL-10 (L), and IL-21 (M) for 7 days. Levels of IgM, IgG and IgA in culture supernatants were determined by ELISA. **(N** and **O)** Memory B cells, isolated by FACS, were stimulated with CD40L in the presence IL-21. Levels of IgG1 (N), IgG2, IgG3, and IgG4 (O) in culture supernatants were determined by ELISA. **(P)** Memory B cells, isolated by FACS, were stimulated with CD40L in the presence or absence of IL-4 and IL-21. Levels of IgE in culture supernatants were determined by ELISA. In Fig. 1A, B, D, G - P bars represent the mean and the standard deviation. Dots represent individual samples for CTL or Age-CTL and technical replicates for M/M. Two-way ANOVA was used in (G – M and P). Mann-Whitney test was used in (N). Student t-test was used in (O). In (G - P), *p<0.05, **p<0.01, ***p<0.001, ****p<0.0001, and ns = not significant (or not marked).

To investigate the impact of T-bet deficiency on human B cells, we first performed flow cytometric immunophenotyping of the patient (P or M/M – referring to genotype Mutant/Mutant)’s peripheral blood mononuclear cells (PBMCs). Frequencies of total B cells in P was greater than in healthy donors (aged 16 – 65 years), but similar to age-matched healthy donors (n=5-8, range 1-7 yrs, mean age 3.7 yrs) (Fig. 1A). This is consistent with the decline in B cell frequency in peripheral blood during the first decade of life (57, 58). Proportions of transitional, naïve, and memory B-cell subsets, as well as of IgG^+^ and IgA^+^ memory B cells, were also similar between P, healthy donors, and age-matched controls (Fig. 1B and C). We investigated whether T-bet regulated production of different classes of Ig by human B cells *in vivo*, as observed for mice (15). Plasma IgA and IgM levels in P were normal, while IgE was elevated (Fig. 1D). Plasma total IgG levels were also higher in P, predominantly due to increased IgG1, and a modest increase in IgG4 (Fig. 1D). By contrast, IgG2 was reduced (Fig. 1D). Consistent with these serological findings, which reflect constitutive production of Ig isotypes by plasma cells, memory B cells in the T-bet-deficient patient contained markedly increased proportions of IgG1^+^ and fewer IgG2^+^ cells than healthy donors (Fig. S1A). We also investigated antigen-specific antibody (Ab) responses of the T-bet deficient patient by determining levels of IgG specific for tetanus toxoid, diphtheria toxoid, and *Haemophilus influenzae b*, which P had been vaccinated against. P had IgG levels against these three vaccines in the normal range of healthy donors (Table S1). In addition, his antibody titer against pneumococcal antigen was also normal (Table S1). Thus, while human T-bet deficiency does not impact the generation of antigen-specific Ab or differentiation of human naïve B cells into memory or plasma cells *per se*, it does skew Ig class switching towards IgG1, IgG4 and IgE, and away from IgG2.

### Altered B-cell receptor repertoire in inherited human T-bet deficiency

We further investigated the consequences of inherited human T-bet deficiency on humoral immunity by analyzing the B cell receptor (BCR) repertoire in P and comparing it to healthy donors. This confirmed the flow cytometric analysis of memory B cells (Fig. 1C), and serum Ig levels (Fig. 1D), which suggested CSR was generally intact in P, however with some differences (Fig. 1E). Specifically, we detected fewer clones expressing IgG2 and more expressing IgG4 in P (Fig. 1E). This difference was particularly marked in comparison with age-matched healthy children and is consistent with the lower levels of serum IgG2 and higher serum levels of IgG4 in P relative to healthy donors. Somatic hypermutation (SHM) was also intact (Fig. 1F). The targeting and nature of SHM within complementarity-determining regions of the Ig H and L chains of human T-bet-deficient total B cells were consistent with B cells from healthy donors. The repertoire of *IGHM*-expressing B cells from P was slightly less diverse than that of healthy donors (Fig. S1B), probably due to the presence of more expanded clones within the IgM repertoire (Fig. S1C). Diversity was also lower for *IGK* and *IGL* in P (Fig. S1B). This may also be due to the presence of some larger clones, based on D20 metrics (Fig. S1D). By contrast, the diversity of IgG and IgA expressed by T-bet-deficient B cells was similar to healthy donors (Fig. S1D). Further analysis indicated that *IGH*, *IGK* and *IGL* gene usage in B cells of P was not different to B cells from healthy donors. However, within IgM-expressing T-bet-deficient B cells, usage of *IGHV3-15, IGHV3-43* and *IGHV7-4-1* genes was higher than in healthy donors (Fig. S1E). Interestingly, IgG^+^ memory B cells of P also displayed higher usage of *IGHV4-34* (20.2% of IgG1 clones) than those from healthy donors (8.5% of clones) (Fig. S1F). Although *IGHV4-34* genes are expressed by autoreactive B cells, the significance of an enriched population of T-bet-deficient IgG^+^ B cells expressing putative self-reactive BCRs is unknown, as there was no clinical or serological evidence of autoantibodies in the patient. Overall, analysis of the BCR repertoire revealed that inherited human T-bet deficiency increased and decreased CSR to IgG4 and IgG2 respectively, but otherwise had no major impact on the generation of diverse polyclonal antibodies or affinity maturation.

### T-bet-deficient B cells differentiate normally into Ig-secreting plasmablasts *in vitro*

We then investigated intrinsic consequences of T-bet deficiency on the differentiation of human B cells into Ig-secreting plasmablasts *in vitro*. Naïve and memory B cells were stimulated with CD40 ligand (CD40L) alone, or together with CpG (CD40L/CpG), different cytokines (59). CD40L/CpG induced marked IgM secretion by naïve B cells, at similar levels for P and healthy donors (Fig. 1G). Stimulation with CD40L/IL-21 resulted in similarly high levels of IgM, and induction of the class-switched isotypes IgG and IgA, in cultures of naïve B cells from healthy donors and from P (Fig. 1H - J). T-bet-deficiency also had no effect on the ability of memory B cells to differentiate into plasmablasts secreting IgM or IgG, regardless of the nature of the stimulus: CD40L/CpG (Fig. 1K), CD40L/IL-10 (60, 61) (Fig. 1L), or CD40L/IL-21 (Fig. 1M). T-bet deficiency slightly increased IgA production by memory B cells stimulated with CD40L/IL-10 (Fig. 1L). Interestingly, T-bet-deficient memory B cells tended to produce larger amounts of IgG than memory B cells from most healthy donors (Fig. 1M). Quantification of IgG subclasses revealed increases in secretion of IgG1 and IgG4 (Fig 1N and O). IgE secretion by memory B cells in response to CD40L/IL-4/IL-21 (62) was also unaffected by inherited T-bet deficiency (Fig. 1P). Thus, human T-bet deficiency does not affect CSR and B-cell differentiation *in vitro* in response to stimulation with cytokines (IL-4, IL-10, IL21), CD40L or TLR agonists. However, similar to findings for serum IgG subclasses, these *in vitro* functional analyses revealed that T-bet deficiency intrinsically alters the capacity of human B cells to differentiate into IgG subclass-specific plasma cells.

### CD21^lo^ B cells are reduced in human T-bet deficiency

We then investigated whether CD19^hi^CD21^lo^ B cells, corresponding to murine ABCs, were affected by inherited human T-bet deficiency. Conventional flow cytometry established that these cells represent ~2.5% of peripheral blood B cells in healthy aged-matched and adult donors but only ~0.5% in T-bet-deficient patient (Fig. S2A). Phenotypic analysis of CD19^hi^CD21^lo^ B cells in healthy donors revealed downregulation of CCR7, CXCR4, CXCR5, and increased expression of CD11c, CXCR3, FCRL5, CD86, CD95, and T-bet relative to CD21^+^ B cells (Fig. S2B). Notably, expression of CXCR3, FCRL5 and T-bet by the residual CD19^hi^CD21^lo^ B cells detected in the T-bet-deficient patient were not upregulated, and CCR7 was not down-regulated, relative to expression levels on CD21^+^ B cells (Fig. S2B).

### T-bet deficiency disrupts generation of the CD11c^+^ subset of CD21^lo^ B cells

**Figure 2.**
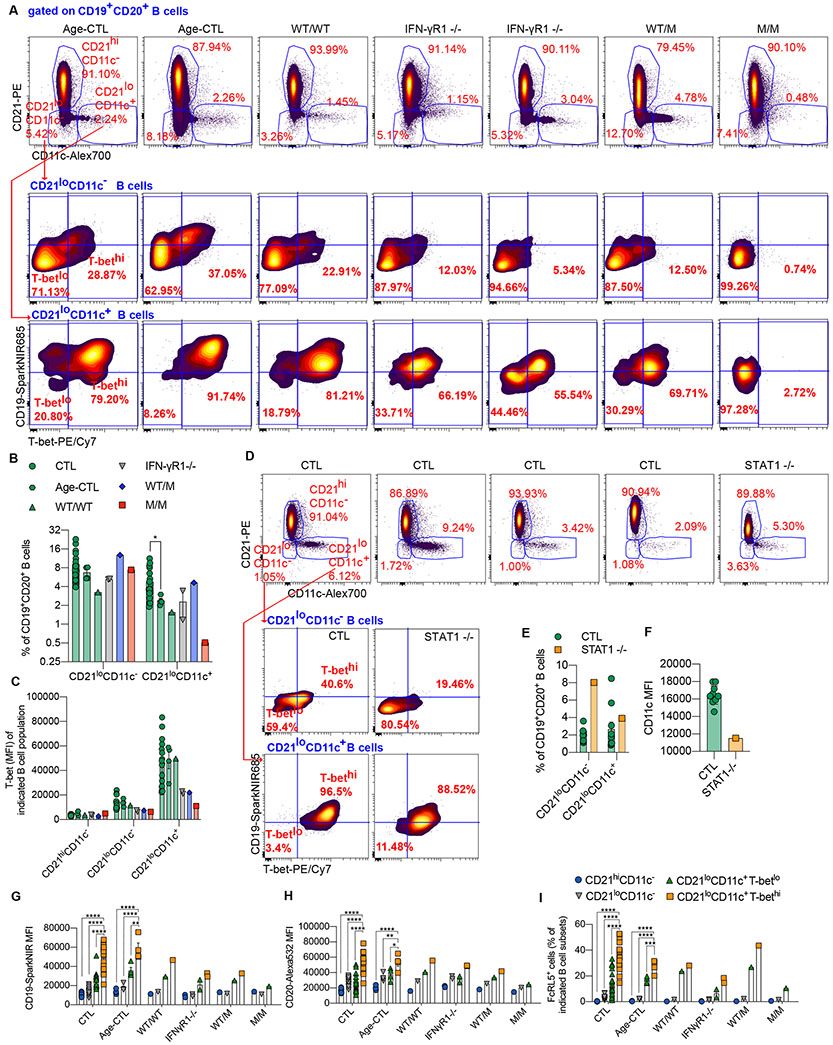
CD21^lo^CD11c^+^ B-cell levels are low in a patient with inherited T-bet deficiency. **(A)** PBMCs from 20 adult controls (CTL), four age-matched controls (Age-CTL), two IFN-γR1-deficient patients (IFN-γR1 ^−/−^), P's healthy brother (WT/WT), heterozygous mother (WT/M), and P (M/M) were analyzed with a 29-color flow cytometry panel focusing on B cells. Surface staining of CD21 and CD11c on CD19^+^CD20^+^ B cells from different individuals, as indicated, is shown. CD21^lo^CD11c^−^ or CD21^lo^CD11c^+^ B cells were gated from CD19^+^CD20^+^ B cells, and their surface expression of CD19 and intracellular expression of T-bet were plotted. (**B**) Percentages of CD21^lo^CD11c^−^ and CD21^lo^CD11c^+^ B cells, as in (A), are shown. **(C)** Mean fluorescence intensity of T-bet of CD21^hi^CD11c^−^, CD21^lo^CD11c^−^, and CD21^lo^CD11c^+^ B cells, as in (A), are shown. (**D**) PBMCs from 10 adult controls (CTL) and a patient with AR complete STAT1 deficiency (STAT1 ^−/−^) were analyzed with the 29-color flow panel as in (A). Surface staining of CD21 and CD11c on CD19^+^CD20^+^ B cells from different individuals, as indicated. CD21^lo^CD11c^−^ or CD21^lo^CD11c^+^ B cells were gated from CD19^+^CD20^+^ B cells, and their surface expression of CD19 and intracellular expression of T-bet were plotted. (**E**) Percentages of CD21^lo^CD11c^−^ and CD21^lo^CD11c^+^ B cells, as in (D). (**F**) Mean fluorescence intensities (MFI) for CD11c expression on CD21^lo^CD11c^+^ B cells gated as in (D), from CTL or the STAT1 ^−/−^ patient. **(G - I)** The expression levels of CD19 (G), CD20 (H), and FcRL5 (I) on CD21^hi^CD11c^−^, CD21^lo^CD11c^−^, CD21^lo^CD11c^+^T-bet^lo^, and CD21^lo^CD11c^+^T-bet^hi^ B cells, as indicated by MFI. In Fig. 2B, C, and E - I, bars represent the mean and the standard deviation. Dots represent individual samples. One-way ANOVA test was performed for (B). Mann-Whitney tests were performed to compare CD21^lo^CD11c^−^, CD21^lo^CD11c^+^T-bet^lo^, and CD21^hi^CD11c^−^ B cells with CD21^lo^CD11c^+^T-bet^hi^ B cells (G - I). In (B, G - I), *p<0.05, **p<0.01, ***p<0.001, ****p<0.0001, and ns = not significant (or not marked).

We extended these findings by performing in-depth analysis of B-cell subsets with a 29-color spectral flow cytometry panel. We focused on expression of CD21 and CD11c on circulating CD3^−^CD56^−^CD19^+^CD20^+^ B cells as CD21^lo^CD11c^hi^ is a common phenotype of murine ABCs and human CD21^lo^ and atypical memory B cells (9, 63-65). CD21^lo^ B cells in healthy donors were heterogeneous, expressing various levels of CD11c (Fig. 2A) (9, 43, 66). CD21^lo^CD11c^+^ B cells from healthy donors expressed higher levels of CD19 and T-bet than corresponding CD21^lo^CD11c^−^ B cells, which mostly lacked T-bet (Fig. 2A - C). CD21^lo^CD11c^+^ B cells were markedly lower in P than in healthy donors (Fig. 2A and B). The residual CD21^lo^CD11c^+^ B cells in peripheral blood of P displayed no detectable expression of T-bet and did not upregulate CD19 (Fig. 2A). CD11c^+^T-bet^+^ B-cells are reduced in IFN-γ deficient mice (34). By contrast, frequencies of CD21^lo^CD11c^+^ B cells in patients with inherited IFN-γR1 deficiency were similar to healthy donors (Fig. 2A and B). However, proportions of CD21^lo^CD11c^+^ B cells with the highest expression of T-bet and CD19 were reduced in patients with IFN-γR1 complete deficiency compared to healthy donors (Fig. 2A and C). This suggests that while IFN-γ signaling, which can induce T-bet in human and murine B cells (6, 7), is not required for the *in vivo* development of CD21^lo^CD11c^+^ B cells in humans, IFN-γ does modulate differentiation of these cells, evidenced by lower expression of CD19 and T-bet on CD21^lo^ B cells from *IFNGR1*-deficient individuals (67).

### IFN-γ, STAT1 and T-bet cooperate to induce the generation of human CD11c^hi^CD21^lo^ B cells *in vivo*

We investigated this aspect further by studying B cells from patients with AR complete STAT1 deficiency, which abolishes signaling via IFN-γ and other STAT1-dependent cytokines (68). Proportions of CD21^lo^CD11c^+^ B cells in AR STAT1-deficient patients were also similar to healthy donors (Fig. 2D and E). Interestingly, STAT1-deficient CD21^lo^CD11c^+^ B cells expressed high levels of CD19, but only intermediate levels of CD11c (CD11c^int^), whereas most of the CD21^lo^CD11c^+^ B cells of healthy donors were CD11c^hi^ (Fig. 2D and F). Similarly, IFN-γR1-deficient CD21^lo^CD11c^+^ B cells expressed lower levels of CD11c compared to CD21^lo^CD11c^+^ B cells from most age-matched controls (Fig. S2C). Interestingly, frequencies of CD21^lo^CD11c^+^ B cells expressing the highest levels of CD11c and T-bet (i.e. CD11c^hi^T-bet^hi^) in AR STAT1 or IFN-γR1 deficiencies were lower than in age-matched and most adult controls (Fig. S2D and E). Strikingly, CD11c^hi^T-bet^hi^ B cells were completely absent in T-bet deficiency (Fig. S2E). Thus, CD21^lo^ cells can be generated in the absence of T-bet, STAT1 or IFN-γR. However, T-bet is strictly required to induce the canonical CD21^lo^CD11c^+^T-bet^hi^ phenotype of this B-cell subset, while STAT1 or IFN-γR are only required to generate the CD11c^hi^T-bet^hi^ subset of human CD21^lo^CD11c^+^ B cells.

### Comprehensive characterization of CD21^lo^CD11c^+^ B cells

Based on spectral flow cytometry, CD21^lo^CD19^hi^CD11c^+^ B cells constitute only a subset of human CD21^lo^ B cells (Fig. 2A). We therefore further explored the nature of human B cell subsets defined by T-bet, CD21, and CD11c expression. In healthy donors, CD21^lo^CD11c^+^ B cells expressed T-bet more strongly than CD21^+^ B cells, but this expression was also heterogeneous, as most CD21^lo^CD11c^+^ B cells lacked T-bet (Fig. S2F). We overlaid CD21^lo^CD11c^+^T-bet^hi^ B cells with their CD21^lo^CD11c^+^T-bet^lo^ counterparts and CD21^hi^CD11c^−^ B cells (Fig. S2F). CD21^lo^CD11c^+^T-bet^hi^ B cells had the highest levels of CD19 and CD20 (Fig. 2G, H; Fig. S2F). We further defined the phenotype of CD21^lo^CD11c^+^T-bet^hi^ B cells relative to CD21^hi^CD11c^−^, CD21^lo^CD11c^−^, and CD21^lo^CD11c^+^T-bet^lo^ B cells. In addition to CD19 and CD20, HLA-DR and FCRL5 were expressed at the highest levels by CD21^lo^CD11c^+^T-bet^hi^ B cells, and at intermediate levels on CD21^lo^CD11c^+^T-bet^lo^ B cells of adult and age-matched healthy donors (Fig. 2I, Fig. S2G). By contrast, CD23, CD24, CD38 and CD40 levels were lowest on CD21^lo^CD11c^+^T-bet^hi^ B cells, intermediate on CD21^lo^CD11c^+^T-bet^lo^ B cells, and highest on CD21^hi^CD11c^−^ B cells (Fig. S2H - K). CD95 was strongly expressed on all CD21^lo^CD11c^+^ B cells, with expression on CD21^lo^CD11c^+^T-bet^lo^ B cells being slightly higher than on CD21^lo^CD11c^+^T-bet^hi^ cells (Fig. S2L). CXCR3 and CD86 were strongly expressed, whereas CXCR4 was only weakly expressed, on CD21^lo^CD11c^+^ cells, but the level of expression of these molecules on CD21^lo^CD11c^+^T-bet^hi^ cells was similar or lower than on CD21^lo^CD11c^+^T-bet^lo^ B cells (Fig. S2M - O). CD21^lo^CD11c^+^T-bet^lo^ B cells also had unusually high levels CD11b, FCRL4, CD10, CD269, CD5, and CD80 generally not observed on their T-bet^hi^ counterparts (Fig. S2P - T). Thus, CD21^lo^CD11c^+^CD19^hi^CD20^hi^ T-bet^+^ B cells constitute a distinct subset of human B cells, with expression of most signature markers being consistent across different age groups, and being T-bet dependent. In contrast, CD21^lo^CD11c^+^T-bet^lo^ B cells appear to be an intermediate precursor of CD21^lo^CD11c^+^T-bet^hi^ B cells or represent a distinct effector B-cell subset.

### Unsupervised analysis confirms the depletion of CD21^lo^CD11c^+^ B cells in T-bet deficiency

**Figure 3.**
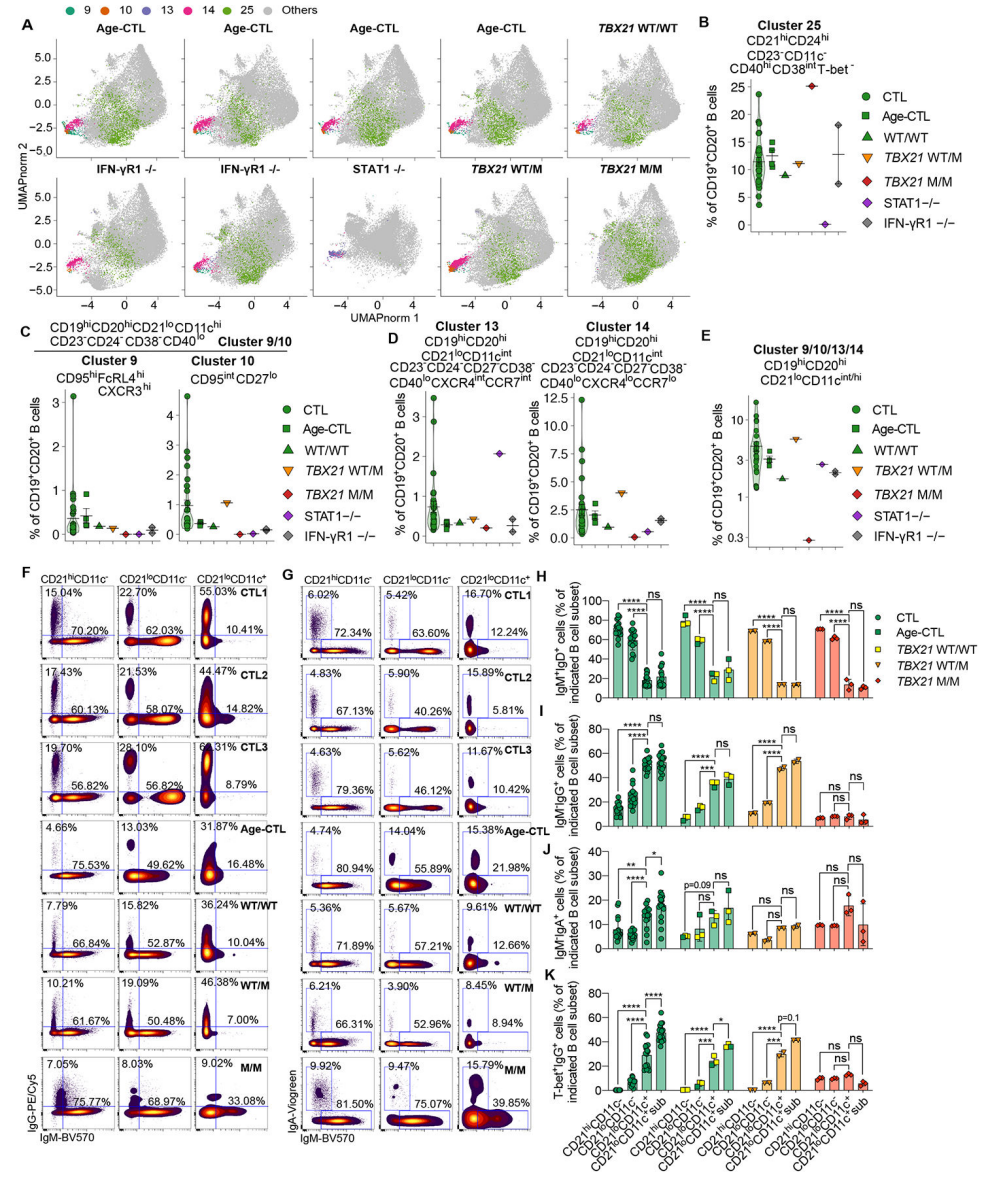
Depletion of some unique subsets of CD21lo B cells in the patient with inherited T-bet deficiency. **(A)** PBMCs from 30 healthy adults (CTL), four age-matched controls (Age-CTL), two IFN-γR1-deficient patients (IFN-γR1 ^−/−^), P's healthy brother (WT/WT), P’s heterozygous mother (WT/M), and P (M/M) were analyzed with a 29-color flow cytometry panel focusing on B cells in two separate experiments. Batch correction was performed with the iMUBAC algorithm and FlowSOM unsupervised clustering was performed. These clusters from age-matched controls (Age-CTL), IFN-γR1-deficient patients (IFN-γR ^−/−^), P (M/M), P's healthy brother (WT/WT), P’s mother (WT/M), and a STAT1-deficient patient (STAT1 ^−/−^) are shown on UMAP graphs. Clusters increased or reduced in P are highlighted. **(B)** Frequency of cluster 25 among B cells. **(C - E)** Frequencies of clusters 9, 10, 13, 14, and combined among B cells. **(F)** PBMCs from P (M/M), adult controls (CTL), age-matched controls (Age-CTL) including P's healthy brother (WT/WT), and heterozygous mother (WT/M) were analyzed with a flow cytometry panel including IgM, IgG, IgA, IgD, and CD71 staining focusing on B cells. Their surface expression of IgM and IgG were plotted. **(G)** Surface expression of IgM and IgA, as in (**F**) were plotted. **(H – K)** Percentages of IgM^+^IgD^+^ (**H**), IgM^−^IgG^+^ (**I**), IgM^−^IgA^+^ (**J**), or T-bet^+^IgG^+^ (**K**) cells, as in (**F**), among indicated subsets of B cells were shown. CD21^lo^CD11c^+^ sub represents CD21^lo^CD11c^+^CD23^−^CD24^−^CD38^−^T-bet^hi^ B cells. In Fig. 3B - E, 3H - K, bars represent the mean and the standard deviation. Dots represent individual samples for CTL or Age-CTL and technical replicates for M/M. One-way ANOVA with multiple comparison tests were performed to compare CD21^hi^CD11c^−^, CD21^lo^CD11c^−^, CD21^lo^CD11c^+^, and CD21^lo^CD11c^+^CD23^−^CD24^−^CD38^−^T-bet^hi^ (CD21^lo^CD11c^+^ sub) B cells against each other in (H – K). In (H - K), *p<0.05, **p<0.01, ***p<0.001, ****p<0.0001, and ns = not significant (or not marked).

To prevent bias introduced by manual gating, and identify other B-cell perturbations in inherited T-bet deficiency, we performed an unsupervised analysis of CD3^−^CD56^−^CD19^+^CD20^+^ B cells with FlowSOM (69, 70). We excluded T-bet from the initial clustering because T-bet-deficient B cells had lower basal levels of T-bet. When B-cell data for all individuals – including healthy donors, IFN-γR1-, STAT1-, and T-bet-deficient patients – were combined, 30 self-organizing clusters were identified (Fig. S3A, S3B; Fig. 3A). Notably, five B-cell clusters were enriched or diminished in the T-bet-deficient patient relative to healthy donors. The percentage of B cells corresponding to cluster 25 (CD21^hi^CD24^hi^CD40^hi^CD23^−^CD11c^−^CD38^int^), probably representing a subset of immature B cells, was higher in P than in adult and age-matched healthy donors but lower in a patient with AR complete STAT1 deficiency (Fig. 3A and 3B). By contrast, four clusters were depleted in P: clusters 13 and 14 (CD19^hi^CD20^hi^CD21^lo^CD23^−^CD24^−^CD11c^int^CD27^−^CD38^−^CD40^lo^) and clusters 9 and 10 (CD19^hi^CD20^hi^CD21^lo^CD23^−^CD24^−^CD11c^hi^CD27^+/−^CD38^−^CD40^lo^) (Fig. 3A, C and D). CD21^lo^CD11c^+^ B cells, including both CD11c^int^ and CD11c^hi^, were therefore the only B-cell subset strictly dependent on T-bet (Fig. 3E). These four clusters (Clusters 9, 10, 13, 14) all expressed high levels of intracellular T-bet (Fig. S3), and the frequencies of each was lowest in the T-bet deficient patient (Fig. 3C - E). Remarkably, these subsets had different developmental requirements. Depletion of clusters 9 and 10, but not clusters 13 or 14, was observed in patients with inherited AR STAT1 deficiency (Fig. 3A, C and D). However, IFN-γR is redundant for the development of cluster 9 (CD19^hi^CD20^hi^CD21^lo^CD23^−^CD11c^hi^CD95^hi^FCRL4^hi^CXCR3^hi^), whereas cluster 10 (CD19^hi^CD20^hi^CD21^lo^CD23^−^CD11c^hi^CD95^int^ CD27^lo^) seemingly required both STAT1 and IFN-γR for their proper development (Fig. 3C). Although signaling via T-bet was indispensable, intact signaling via IFN-γR or STAT1 was not required for the generation of CD21^lo^CD11c^int^ B cells corresponding to clusters 13 and 14 (Fig. 3D). This is consistent with our earlier observation that most CD21^lo^CD11c^+^ B cells in STAT1-deficient individuals expressed intermediate levels of CD11c (Fig. 2F).

### IgA- and IgG-expressing B cells are enriched in CD21^lo^CD11c^+^ B cells, which correlates strongly with high T-bet expression

To evaluate expression of Ig isotypes by CD21^lo^CD11c^+^ and other B cell subsets, we integrated the surface expression of BCRs into a 30-color spectral flow phenotyping panel. Frequencies of CD21^lo^CD11c^+^ B cells in inherited human T-bet deficiency were significantly lower than in adult and age-matched controls (Fig. S4A). Frequencies of unswitched IgM^+^IgD^+^ B cells were significantly reduced in CD21^lo^CD11c^+^ B cells from adult, age-matched healthy donors and T-bet deficiency compared to CD21^hi^CD11c^−^ and CD21^lo^CD11c^−^ B cell subsets (Fig. 3F - H). By contrast, frequencies of IgG^+^ or IgA^+^ switched B cells were significantly increased in CD21^lo^CD11c^+^ B cells among healthy donors, but not T-bet deficient P, relative to these other B-cell subsets (Fig. 3F, 3G, 3I and 3J). These IgG^+^ or IgA^+^ switched CD21^lo^CD11c^+^ B cells did not express the surface memory marker CD27 (Fig. S4B). This was in striking contrast to IgG^+^ or IgA^+^ CD21^hi^CD11c^−^ B cells that were mostly CD27^hi^ (Fig. S4B). Most IgG^+^ or IgA^+^ CD21^lo^CD11c^+^ B cells from healthy donors displayed the highest expression of T-bet amongst all B-cell subsets examined (Fig. S4C - E and Fig. 3K). CD71 can delineate early activated CD20^hi^ B cells from resting naïve B cells (71). CD71^+^CD20^hi^ B cells share some phenotypic similarity with CD21^hi^CD11c^−^ cells and CD21^lo^CD11c^+^ B cells such as high CD80 expression (Fig. S4F). Interestingly, frequencies of CD71^hi^CD80^hi^ cells were slightly enriched in CD21^lo^CD11c^+^ B cells and drastically increased in the remaining few CD21^lo^CD11c^+^ B cells in T-bet deficiency (Fig. S4F and G). Therefore, IgG^+^ or IgA^+^ B cells are enriched in CD21^lo^CD11c^+^ B cells in healthy donors but not T-bet deficiency. Expression of IgA and IgG on these CD21^lo^CD11c^+^ B cells correlate strongly with high T-bet expression, collectively suggesting a critical role of T-bet in the development of this distinct subset of B cells.

### Single-cell proteotranscriptomics of CD21^lo^ B cells reveals the complete depletion of a distinct subset of human CD21^lo^ B cells in inherited T-bet deficiency

**Figure 4.**
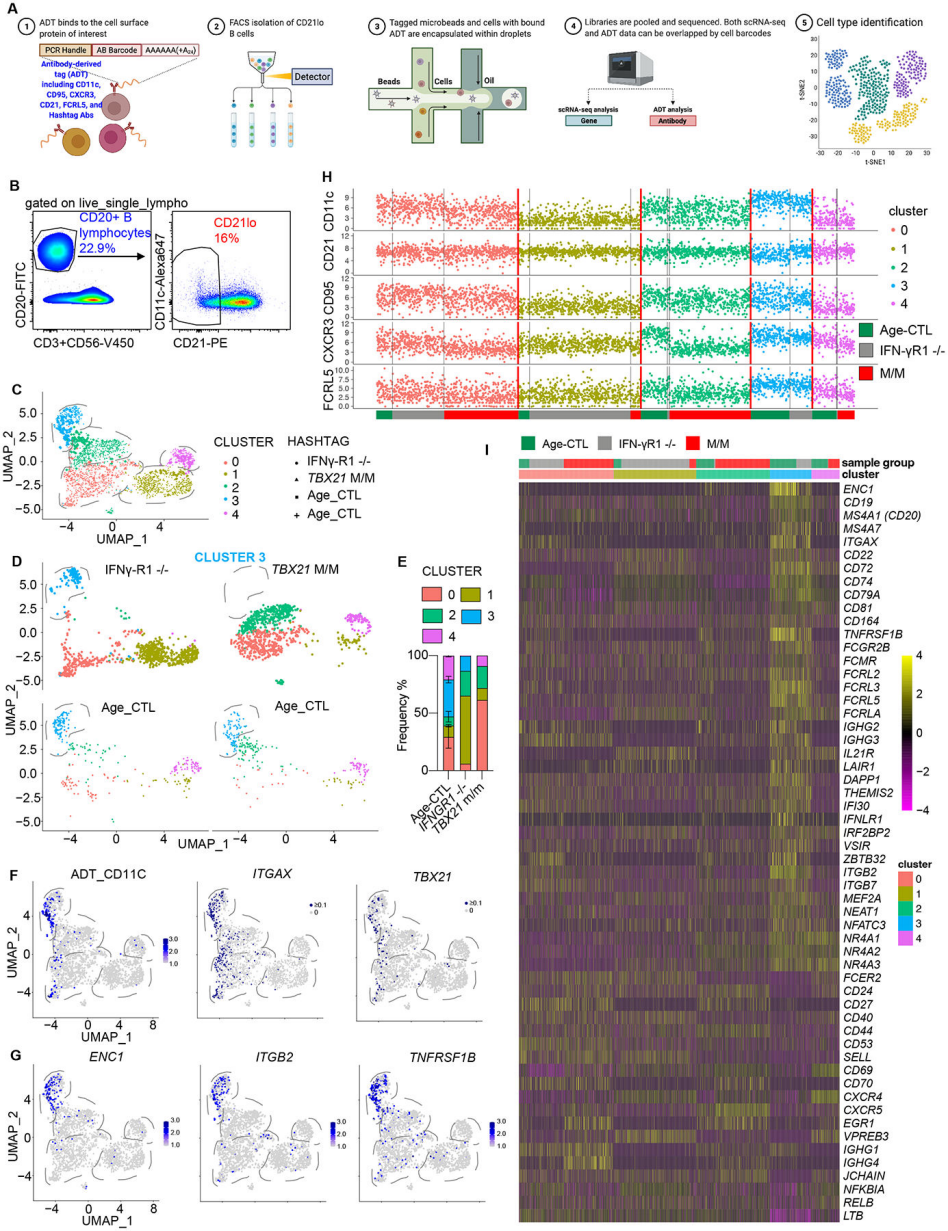
CITE-seq of CD21^−^ B cells from a patient with inherited human T-bet deficiency. **(A)** A schematic diagram of the experimental design. **(B)** PBMCs from the indicated individuals were labeled with hashtag Abs and oligonucleotide-conjugated anti-CD11c, anti-CD21, anti-CD95, anti-CXCR3, anti-FcRL5 Abs. CD3^−^CD56^−^CD20^+^CD21^lo^ B cells were isolated by FACS. **(C)** CD3^−^CD56^−^CD20^+^CD21^−^ B cells, as in (**B**), were subjected to CITE-seq. Cells from two Age-CTL, one IFN-γR1 ^−/−^ patient, and P, which had similar patterns of housekeeping gene expression, were subjected to dimensionality reduction by UMAP based on their transcriptome. **(D)** Four individual samples from the pool, as in (**C**), were split on the basis of their hashtags. **(E)** Frequencies of each cluster of CD21^−^ B cells from two Age-CTLs, one IFN-γR1 ^−/−^ patient, and P, as in (C and D). **(F)** Cells expressing CD11c surface protein detected by CITE-seq (ADT_CD11c), and cells expressing *ITGAX* or *TBX21* were highlighted in UMAP plots. **(G)** Cells expressing *ENC1*, *ITGB2*, and *TNFRSF1B* were highlighted in UMAP plots. **(H)** Protein levels for CD11c, CD21, CD95, CXCR3, and FcRL5, as determined by CITE-seq, grouped by transcriptome-defined clusters and sample groups. **(I)** Heat map showing the scaled expression levels of a selection of genes differentially regulated in cluster 3 B cells as in (C and D). In Fig. 4E, bars represent the mean and the standard deviation. Dots represent individual samples.

To test if the apparent absence of the CD21^lo^CD11c^+^CD23^lo^CD24^lo^CD38^lo^ B-cell subset in T-bet deficiency may result from the lack of surface markers potentially regulated by T-bet, we performed single-cell (sc) proteotranscriptomic profiling of CD21^lo^ B cells. PBMCs from age-matched healthy donors, IFN-γR1-deficient and T-bet-deficient patients were labeled individually with oligonucleotide (OGN)-barcoded Hashtag Abs and OGN-conjugated TotalSeq Abs against CD21, CD11c, CD95, CXCR3, and FCRL5. To avoid epitope competition, different clones of anti-CD21 and CD11c Abs that recognize unique epitopes, were used for flow and TotalSeq purposes (Fig. S5A and B). Cells were pooled, FACS-sorted as live CD20^+^CD21^lo^ B cells, followed by CITE-seq (cellular indexing of transcriptomes and epitopes by sequencing) and sc-VDJ sequencing (Fig. 4A and B, Fig. S5C). We analyzed 328 and 273 CD21^lo^ cells from two age-matched controls, 913 CD21^lo^ cells from the IFN-γR1-deficient patient, and 937 CD21^lo^ cells from P. Based on unbiased automated clustering, CD21^lo^ B cells from these four individuals formed five distinct clusters - 0, 2, 3, and 4 (Fig. S5D, Fig. 4C and Data file S1) - each with a unique transcriptomic signature (Fig. S5D). Cluster 0 corresponded to memory B cells, with high levels of *CD27*, *CD99*, *LTB*, and *CD53*; cluster 1 corresponded to transitional B cells with high levels of *IGHM*, *IGHD*, *ISG20*, *IL4R* and *CCR7*; cluster 2 had the highest levels of *CXCR5* and *SOCS3*; cluster 3 corresponded to CD21^lo^CD11c^hi^T-bet^+^ B cells, defined by spectral flow cytometry, as shown by high expression of *CD19*, *MS4A1* (CD20), *ITGAX* (CD11c), *FCRL2*, *FCRL3*, and *FCRL5*; cluster 4 corresponded to cells with high levels of AP-1 subunits as well as *CD69*, *CD9*, *CD38* and *CD55* (Fig. S5D). Strikingly, the T-bet-deficient patient was completely devoid of cluster 3, which was present in age-matched controls (Fig. 4D and E). Consistent with cytometric data, cluster 3 B cells were reduced – but still detectable – in IFN-γR1-deficient patients (Fig. 4E). Therefore, CD21^lo^CD11c^+^ B cells form a distinct T-bet-dependent B-cell subset with a distinguishable pattern of signature gene expression that is consistent with the unique pattern of surface marker expression.

### Characterization of CD21^lo^CD11c^+^ B cells by single cell proteotranscriptomics

Transcripts of *TBX21*, encoding T-bet, were barely detectable by CITE-seq. However, most cells containing *TBX21* mRNA belonged to cluster 3 corresponding to CD21^lo^CD11c^+^ B cells (Fig. 4F). These B cells had the highest levels of CD11c protein and mRNA (*ITGAX*, Fig. 4F). *ENC1*, *ITGB2* (encoding CD18), *TNFRSF1B* (encoding TNFR2), *FCRL5*, *CD72*, *FCRL2* and *FCRL3* mRNA, and FCRL5 protein were also highest in this subset (Fig. 4G, 4H, Fig. S5E - H). Of note, *CXCR3* mRNA expression was low in all clusters suggesting high mRNA expression of *CXCR3* may precede the differentiation of these B cell subsets (Fig. S5E). Importantly, higher expression of *ENC1* in CD21^lo^ B cells was confirmed by quantitative PCR (Fig. S5I). By contrast, CD21^lo^CD11c^+^ B cells had low levels of *FCER2* (CD23a, low-affinity IgE receptor), *CD24*, *CD27*, *ITGAM* (CD11b), *SELL* (CD62L) and *LTB* (Fig. S5J). *TFRC* or *CD71*, the mRNA expression of which is elevated in human early activated B cells (71), was not increased in CD21^lo^CD11c^+^ B cells (Fig. S5K). The pattern of expression of these genes by CD21^lo^CD11c^+^ B cells in healthy donors was consistent with the levels of the corresponding proteins in the T-bet-dependent subsets identified by flow cytometry (Fig. 3 and Fig. S2). We identified additional genes differentially expressed between CD21^lo^CD11c^+^ B- and other CD21^lo^ B-cell clusters. *MS4A7*, *CD22*, *CD72*, *CD74*, *CD79A*, *CD81*, *CD164*, *FCGR2B*, *FCMR*, *FCRLA*, *IL21R*, *ITGB2*, *ITGB7*, *NFATC3*, *NR4A1*, *NR4A2*, and *NR4A3*, and the HLA class II genes including *HLA-DRB1*, *HLA-DPB1*, *HLA-DPA1* and *HLA-DQA1*, were significantly higher, while *CD44*, *CD53*, *CD69*, *CD70*, *CXCR4*, *CXCR5*, *NFKBIA*, and *RELB* were significantly lower, in CD21^lo^CD11c^+^ B cells (cluster 3) compared to the other clusters (Fig. 4I, Fig. S5L, Data file S1). Higher levels of FcR, complement receptor 4, NR4A family, and HLA class II genes in CD21^lo^CD11c^+^ B cells suggest these cells function in antigen-presentation (72), cognate T-B cell interactions, and peripheral tolerance (73).

### Normal CSR and SHM in CD21^lo^CD11c^+^ B cells

**Figure 5.**
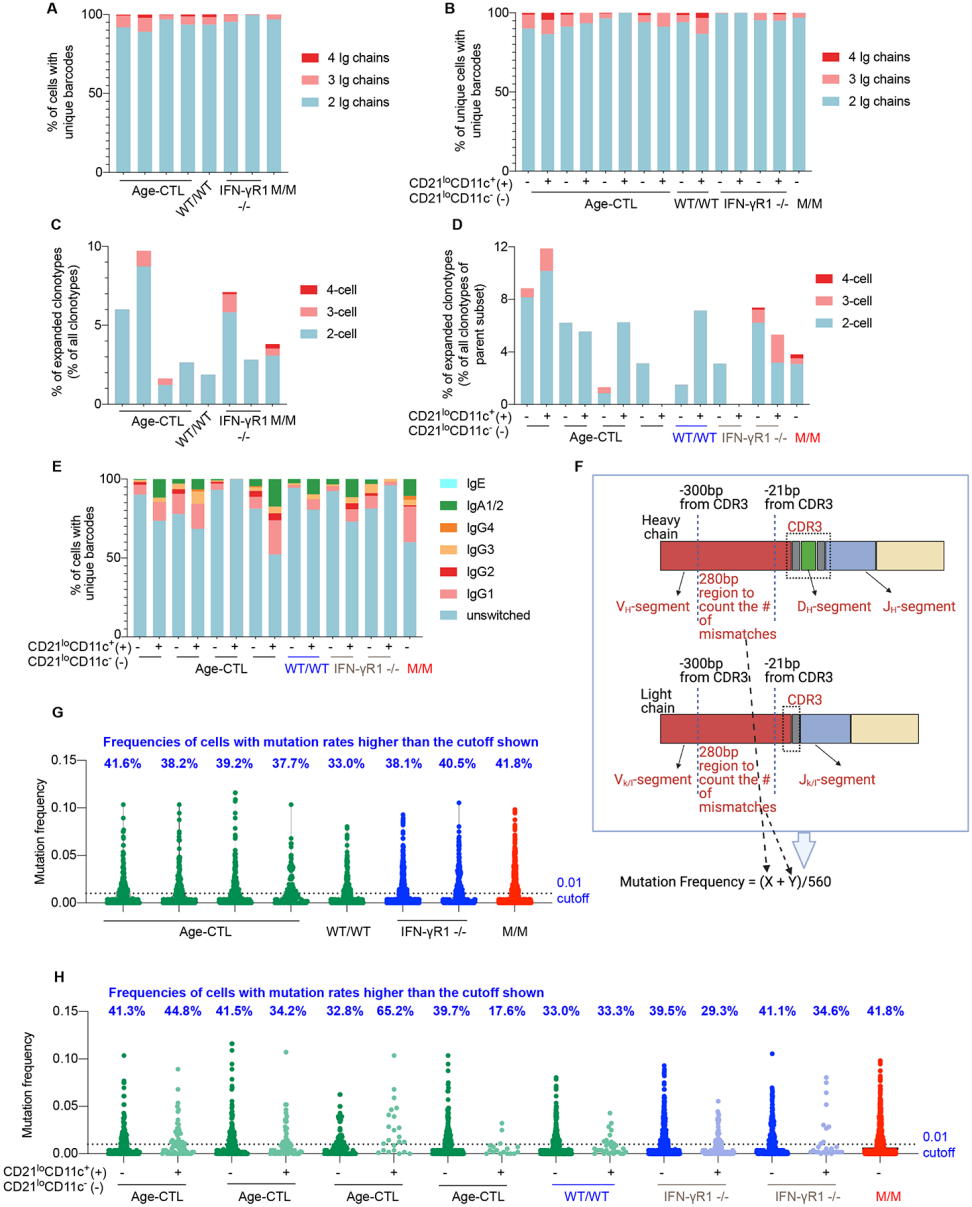
CSR and somatic hypermutation in T-bet-dependent CD. (**A**) Single-cell VDJ sequencing was performed jointly with CITE-seq. Frequencies of CD21^lo^ cells with more than one heavy or light chain are shown. (**B**) Frequencies of cells with more than one heavy or light chain within CD11c^+^ CD21^lo^ B cells (+) or CD11c^−^ CD21^lo^ (−) cells. (**C**) Frequencies of clonotypes common to at least 2 cells in each individual CD21^lo^ sample. (**D**) Frequencies of clonotypes common to at least 2 cells within CD11c^+^ CD21^lo^ B cells (+) or CD11c^−^ CD21^lo^ (−) cells. (**E**) Frequencies of cells that were unswitched or class-switched to IgE (IGHE), IgA1 (IGHA1) or IgA2 (IGHA2, combined as IgA1/2), IgG1 (IGHG1), IgG2 (IGHG2), IgG3 (IGHG3), or IgG4 (IGHG4) among CD21^lo^ cells. (**F**) Somatic hypermutation was analyzed as shown in this schematic diagram. Briefly, the numbers of mutations relative to the predicted germline sequence within a 280-nucleotide region (−21 to −300 bp from the start of CDR3 region) of each heavy and light chain were counted. The total number of mutations for both heavy and light chains for each given cell was divided by the total number of nucleotides counted, to calculate the mutation frequency. (**G**) Mutation frequency of each CD21^lo^ B cell from the indicated individuals. The frequencies of cells with mutation rates greater than 1% are highlighted. (**H**) Mutation frequency of each CD11c^+^ CD21^lo^ B cells (+) or CD11c^−^ CD21^lo^ (−) B cells from the indicated individuals. The frequencies of cells with mutation rates greater than 1% are highlighted. In Fig. 5A - E, bars represent values of each individual sample. In Fig. 5G and H, dots represent values for individual cells.

We then analyzed sc-VDJ sequencing data. A few B cells expressed >1 Ig H or L chain gene, consistent with recent reports (74, 75). The frequencies of cells with 3-4 consensus Ig chains were similar in P and age-matched controls (Fig. 5A). The frequencies of cells expressing >1 Ig H or L chain gene were similar for CD21^lo^CD11c^+^ B cells and other cells (Fig. 5B). We then assessed the clonality of each sample. Cells with identical sequences for the junctional region, the CDR3 region, or both IgH or IgL chains were considered to be of the same clonotype. Most B cells were unique. However, 1-10% of clonotypes were common to >1 CD21^lo^ B cell. The frequency of expanded clonotypes among CD21^lo^CD11c^+^ B cells did not differ between the T-bet-deficient patient and healthy donors (Fig. 5C). Frequencies of expanded clonotypes were similar among CD21^lo^CD11c^+^ B cells from healthy donors and IFN-γR1-deficient patients (Fig. 5D). Consistent with enriched IgG- and IgA-expressing B cells in CD21^lo^CD11c^+^ B cells (Fig. 4), single-cell studies showed that the frequencies of IgG- and IgA1/2-switched B cells among CD21^lo^CD11c^+^ B cells of most healthy donors were higher than those among other CD21^lo^ B-cell counterparts (Fig. 5E). We also assessed SHM frequency in a 280-nucleotide (nt) region upstream from the CDR3 site at single-cell level, by comparing the assembled sequences with their predicted germline sequences (76, 77) (Fig. 5F). SHM frequencies ranged from 0 to 10%. CD21^lo^ B cells from T-bet-deficient or IFN-γR1-deficient patients displayed similar levels of SHM to CD21^lo^ B cells of age-matched controls (Fig. 5G). CD21^lo^CD11c^+^ B cells from healthy donors and IFN-γR1-deficient patients had SHM rates similar to those in other CD21^lo^ B-cell counterparts (Fig. 5H). Thus, CD21^lo^CD11c^+^ B cells had levels of clonal expansion, CSR and SHM similar to those of other CD21^lo^ B cells. However, IgG- and IgA-enriched B cells appear accumulate in CD21^lo^CD11c^+^ B cells.

### Signaling via TLR, BCR and IFN-γ or IL-27 induce T-bet^hi^CD19^hi^CXCR3^+^ B cells *in vitro*

**Figure 6.**
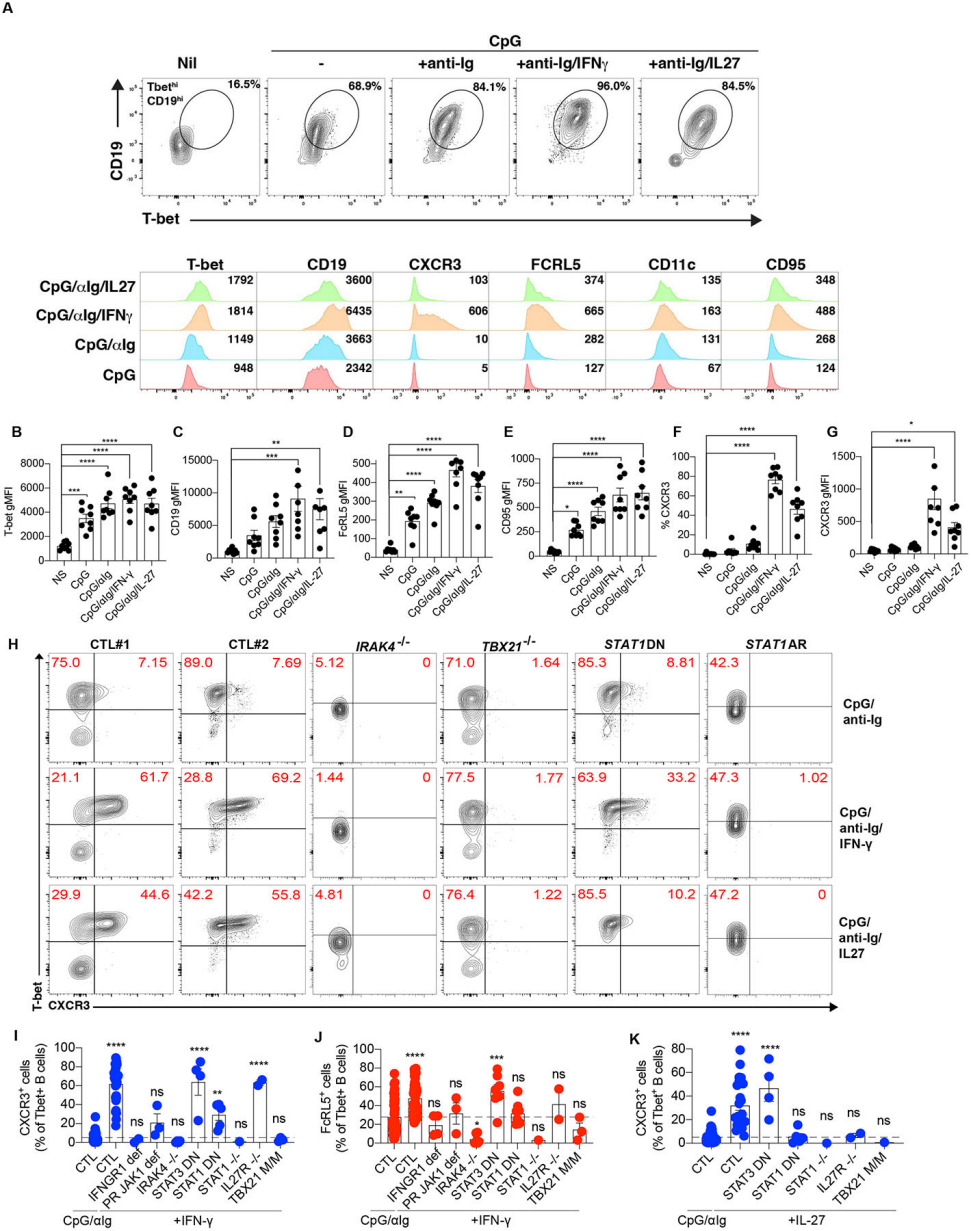
T-bet functions in a B cell-intrinsic manner to induce the generation of T-bet^+^ B cells *in vitro*. **(A)** Naïve B cells were purified from the peripheral blood of healthy donors (*n*=8) and cultured *in vitro* in medium alone (NS: non-stimulated), or with CpG_2006_, CpG_2006_ plus F(ab’)_2_ fragments of goat anti-IgM/G/A Ab (CpG/aIg), CpG/aIg and IFN-γ (CpG/aIg/IFNγ), or CpG/aIg and IL-27 (CpG/aIg/IL-27). After 3.5 days, B cells were harvested, stained for the surface expression of CD19, CD95, CXCR3 FCRL5, and then for the intracellular expression of T-bet. Expression of T-bet and CD19 in B cells under indicated conditions from a healthy donor was plotted. Expression of T-bet, CD19, CXCR3, FCRL5, CD11c, and CD95 were shown. (**B - G**) Viable cells were then analyzed, to determine the level of expression (geometric mean fluorescence intensity [gMFI]) as in (A). The expression (gMFI) of (B) T-bet, (C) CD19, (D) FCRL5, (E) CD95 and (G) CXCR3 on CD19^+^T-bet^+^ B cells, and the proportions of T-bet^+^ B cells co-expressing CXCR3 (F) were also determined. (**H**) Naïve B cells purified from healthy donors or patients with pathogenic variants of *IFNGR1*, *IL27R*, *JAK1*, *IRAK4*, *STAT3* DN, *STAT1* (both AR and DN), or *TBX21* were cultured *in vitro* with anti-IgM/G/A Ab (CpG/aIg), CpG/aIg and IFN-γ (CpG/aIg/IFNγ), or CpG/aIg and IL-27 (CpG/aIg/IL-27) for 3.5 days. Expression of surface CXCR3 and intracellular expression of T-bet in B cells from indicated donors were plotted. **(I** and **J)** The proportions of CD19^+^T-bet^+^ B cells expressing (I) CXCR3 or (J) FCRL5 under anti-IgM/G/A Ab (CpG/aIg) or CpG/aIg and IFN-γ (CpG/aIg/IFNγ) conditions as in (H) were determined. **(K)** The proportions of CD19^+^T-bet^+^ B cells expressing CXCR3 under anti-IgM/G/A Ab (CpG/aIg) or CpG/aIg and IL-27 (CpG/aIg/IL-27) conditions as in (H) were determined. Fig. 6B - G, I - K show the mean ± standard error. Dots represent individual samples for CTL or Age-CTL and technical replicates for M/M. One-way ANOVA was used to compare each set of stimulation conditions with non-stimulated (NIL) conditions in (B – G). One-way ANOVA was used to compare control cells stimulated with anti-IgM/G/A Ab (CpG/aIg) with cells stimulated with CpG/aIg/IFNγ or CpG/aIg/IL-27 (I – K). *p<0.05, **p<0.01, ***p<0.001, ****p<0.0001, and ns = not significant (or not marked). ND: not done

The lack of CD21^lo^CD11c^+^ B cells in P demonstrates an indispensable role for T-bet in generating and/or maintaining these cells *in vivo*, but it remains unknown whether the requirement for T-bet in this process is B cell intrinsic or extrinsic. We addressed this by investigating the ability of naïve B cells from healthy donors to differentiate into T-bet-expressing B cells *in vitro*. CpG stimulation induced expression of T-bet in, and increased CD19 on, human naïve B cells (Fig. 6A) (6, 7, 78). The proportion of T-bet^hi^CD19^hi^ B cells was further enhanced by costimulation with anti-Ig (αIg). Addition of IFN-γ or IL-27 modestly increased expression of T-bet and/or CD19 by CpG/αIg-primed naïve B cells (Fig. 6A-C). Extended phenotypic analysis of *in vitro*-derived T-bet^hi^CD19^hi^ B cells revealed that CpG/αIg-stimulation induced stronger FCRL5, CD95, and CD19 expression than CpG alone (Fig. 6B - D). Notably, addition of IFN-γ or IL-27 to CpG/αIg-stimulated naïve B cells led to further increases in expression of FCRL5, CD95, and CD19 (Fig. 6A, D, E). Furthermore, CXCR3 was induced on 30 to 65% of T-bet^hi^CD19^hi^ B cells following culture with CpG/αIg and either IFN-γ or IL-27, but on fewer than <10% of cells in response to CpG/αIg alone *in vitro* (Fig. 6F, G). Thus, these in vitro culture conditions provide a model to determine the intrinsic molecular requirements for generating T-bet^hi^CD19^hi^ B cells from naïve B cell precursors.

### T-bet functions in a B cell-intrinsic manner to induce the generation of CD21^lo^CD11c^+^ B cells

We then subjected naïve B cells from P, and patients with autosomal dominant (AD) partial IFN-γR1 deficiency (79-81), dominant negative (DN) STAT3 deficiency (82), AD or AR STAT1 deficiency (82-84), AR complete IL-27R deficiency (unpublished), partial recessive JAK1 deficiency (unpublished), or AR IRAK4 deficiency (67, 85) to these culture conditions. As expected, IRAK4 deficiency completely abolished induction of T-bet in B cells stimulated with CpG alone or together with other stimuli, establishing a requirement for TLR signaling in generating T-bet-expressing B cells in *vitro* (Fig. 6H). In contrast, T-bet was induced in T-bet-, AD STAT1, AR STAT1, AR IL27R- and AD IFN-γR1-deficient naïve B cells stimulated with CpG/αIg, CpG/αIg/IFN-γ or CpG/αIg/IL-27 (Fig. 6H). However, T-bet-deficient B cells had lower levels of T-bet than naïve B cells from most healthy donors (Fig. 6H), suggesting T-bet promotes its own expression. Neither IFN-γ nor IL-27 induced CXCR3 expression on naïve B cells from patients with AD IFN-γR1 deficiency or AR complete IL-27R-deficiency, respectively (Fig. 6H- K). The ability of these cytokines to induce CXCR3 on CpG/αIg-stimulated naïve B cells was also reduced by AD STAT1 deficiency and completely abolished by AR STAT1 deficiency but was unaffected by DN mutations in *STAT3* (Fig. 6H - K). Similar results were obtained for upregulation of CD19 and FCRL5 expression mediated by IFN-γ, inasmuch that this was abolished by bi-allelic mutations in *TBX21*, *STAT1*, and impaired by DN mutations in *IFNGR1* or *STAT1* (Fig. 6H - K). The role for STAT1 in this process was also revealed by the ability of JAK inhibitors to prevent the generation of T-bet^hi^CXCR3^+^ B cells from naïve B cells from healthy donors *in vitro* (67). These results establish that signals mediated by TLRs and the BCR in the presence of cytokine inputs initiate the differentiation of naïve B cells into T-bet^+^CD19^hi^ B cells independently of T-bet. However, T-bet is strictly required for the generation of T-bet^+^CD19^hi^CXCR3^+^FCRL5^hi^ B cells *in vitro*.

### Chromatin accessibility of B cells is altered in inherited T-bet deficiency

**Figure 7.**
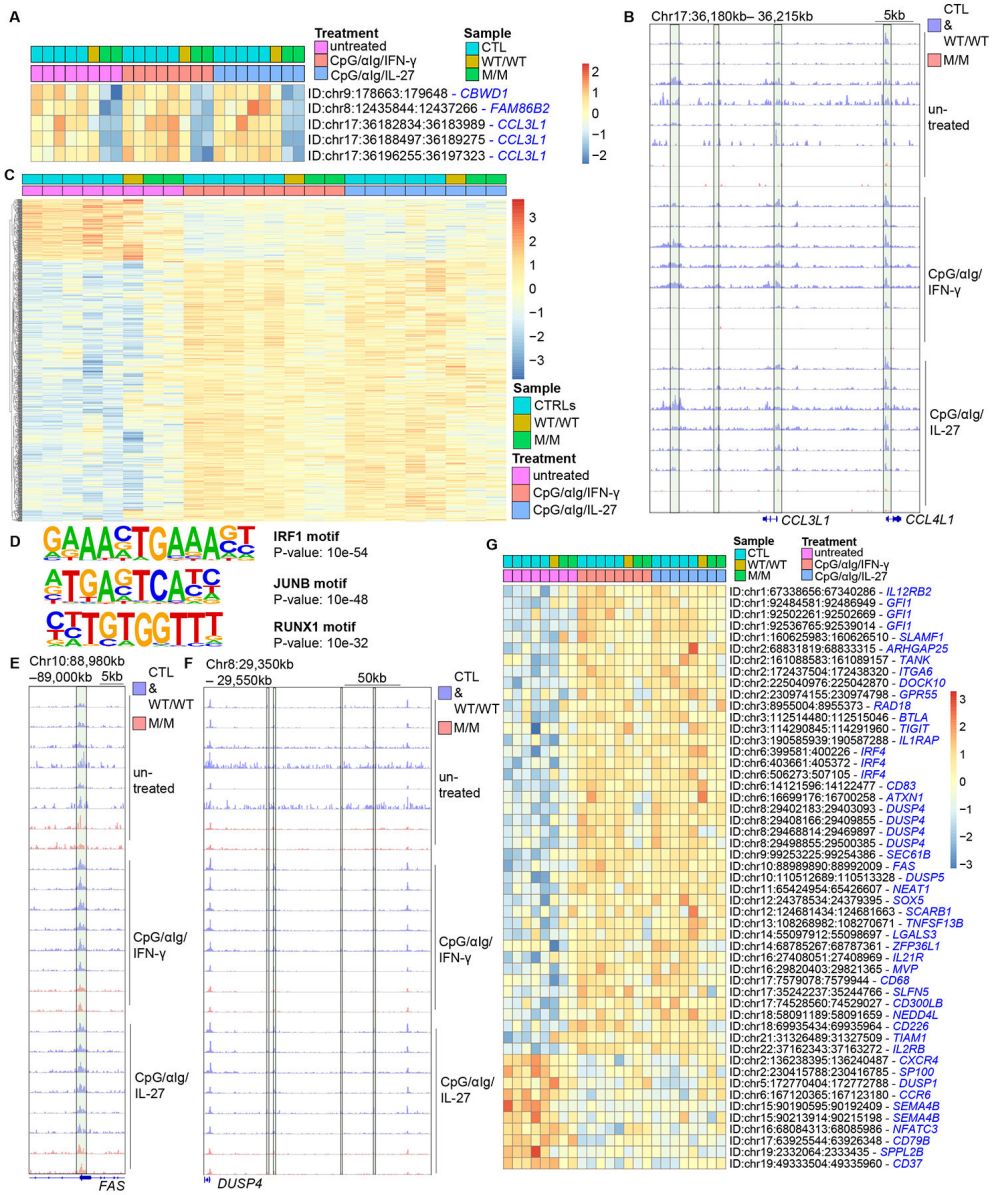
A unique epigenetic landscape determined by T-bet programs B-cell differentiation *in vitro*. (**A**) A heat map showing five loci at which chromatin accessibility differed between controls (CTL) and P (M/M) in the absence of stimulation and in response to aIg+CpG+IFN-γ and aIg+CpG+IL-27. (**B**) Chromatin accessibility of the *CCL3L1* and *CCL4L1* loci, at which the chromatin accessibilities of three regions differed between controls (CTL) and P (M/M) in the absence of stimulation and in response to aIg+CpG+IFN-γ and aIg+CpG+IL-27. (**C**) A heat map showing 902 T-bet-dependent loci, the chromatin accessibilities of which were differentially regulated in control cells, but not significantly different in the cells of P, in response to both aIg+CpG+IFN-γ and aIg+CpG+IL-27. **(D)** DNA motifs most significantly enriched in the 902 T-bet-dependent loci, the chromatin accessibilities of which were differentially regulated in control cells as in (C). (**E** and **F**) Chromatin accessibility of *FAS* (E) and *DUSP4* loci (F), that were differentially regulated in response to aIg+CpG+IFN-γ and aIg+CpG+IL-27 in control B cells but not in T-bet-deficient B cells. (**G**) A heat map showing a selection of 51 loci from the list, as in (C), the chromatin accessibilities of which were differentially regulated in control cells, but not in T-bet-deficient cells, in response to both aIg+CpG+IFN-γ and aIg+CpG+IL-27.

To explore mechanisms by which T-bet controls the lineage determination of T-bet-expressing B cells in humans, naive B cells from healthy donors and P were stimulated with CpG/αIg in the presence of IFN-γ or IL-27. Cells were subjected to Omni-ATAC-seq for the genome-wide investigation of chromatin accessibility (86). In the absence of stimuli, chromatin accessibility differed between B cells from healthy donors and P for only 33 loci (Fig. S6A). Only five of these loci also presented differences in chromatin accessibility between naïve B cells from healthy donors and P in response to stimulation with CpG/αIg/IFN-γ or CpG/αIg/IL-27 (Fig. 7A). Three of these were encompassed by the *CCL3L1* locus, and their chromatin was in the closed configuration in T-bet deficiency, with another proximal locus within *CCL4L1* following the same trend (Fig. 7A and Fig. 7B). This suggests that T-bet-deficient B cells are less poised to secrete chemokines required for T-cell recruitment and are therefore less likely to receive sufficient T-cell help (87). Following *in vitro* stimulation with CpG/αIg/IFN-γ, chromatin accessibility differed significantly between B cells from healthy donors and P for 2391 loci. For 139 loci, chromatin accessibility differed significantly between B cells from healthy donors and those of P following CpG/αIg/IL-27 stimulation, and 50 of these loci overlapped with those displaying differential chromatin accessibility after CpG/αIg/IFN-γ stimulation (Fig. S6A). This finding suggests that IFN-γ and IL-27 stimulate B cells through a common mechanism, probably involving T-bet, but that IFN-γ is the more potent stimulus, consistent with larger proportions of T-bet^+^CXCR3^+^FCRL5^+^ cells induced by IFN-γ from CpG/αIg-stimulated naïve B cells (Figure 6E and F). Thus, inherited human T-bet deficiency leads to changes in chromatin accessibilities of targets common to stimuli known to induce T-bet in B cells.

### Changes in the epigenetic landscape determined by T-bet program B cell differentiation *in vitro*

We analyzed epigenetic changes governed by T-bet in B cells by first studying changes in chromatin accessibility in activated B cells from healthy donors. Chromatin accessibility was upregulated at 2017 loci and downregulated at 461 loci in response to CpG/αIg/IFN-γ in B cells from healthy donors relative to unstimulated B cells. 89% (2208) of these 2478 differentially regulated loci remained unaltered in CpG/αIg/IFN-γ-stimulated T-bet-deficient B cells (Fig. S6B). Chromatin accessibilities of 1184 loci were upregulated, and those of 352 loci were downregulated in B cells from healthy donors in response to CpG/αIg/IL-27. As for CpG/αIg/IFN-γ stimulation, most (89%) remained unaltered by CpG/αIg/IL-27 in T-bet-deficient B cells (Fig. S6B). Thus, the majority of epigenetic changes caused by stimuli that induce T-bet in human B cells were T-bet-dependent. These 2208 (CpG/αIg/IFN-γ) and 1363 (CpG/αIg/IL-27) loci therefore represent the landscape of a T-bet-dependent chromatin signature driving lineage determination in T-bet-expressing B cells (Fig. S6C). Notably, 902 loci (66% of T-bet-dependent targets induced by CpG/αIg/IL-27) overlapped with those induced by CpG/αIg/IFN-γ stimulation (Fig. 7C, Fig. S6C, and Data file S2). DNA binding motifs for IRF1, JUNB, and RUNX1 were most significantly enriched in these 902 shared loci (Fig. 7D and Data file S2). Notably, enrichment in DNA binding motifs of IRF1, a crucial transcription factor downstream of IFN-γ-dependent response in humans (88, 89), suggests T-bet provides permissive environment for binding of IRF1 to IFN-γ- and IL-27-dependent targets in human B cells. Chromatin at the *FAS*, *IL21R*, *SEC61B*, *DUSP4*, *DAPP1*, and *SOX5* loci, which are all were strongly expressed by CD21^lo^CD11c^+^ B cells, was in an open configuration, whereas that at the *CD79B* and *CXCR4* loci, which are weakly expressed in CD21^lo^CD11c^+^ B cells, was closed by both stimuli in a T-bet-dependent manner (Fig. 7E - G, Fig. S6D). These investigations revealed many new T-bet-dependent epigenetic targets. For example, chromatin was in an open configuration at three loci within *IRF4* and three within *GFI1* in B cells from healthy donors, but not in those of P; chromatin accessibility at these loci was increased by CpG/αIg/IFN-γ and CpG/αIg/IL-27, whereas it was decreased at the *SEMA4B*, *CCR6*, and *CD37* loci, by both stimuli, in a T-bet-dependent manner (Fig. 7G and Fig. S6E). These findings suggest that T-bet poises the cells for differentiation into T-bet-expressing B cells by creating a permissive chromatin environment facilitating the efficient differentiation of human CD21^lo^CD11c^+^ B cells (90).

## Discussion

We report that while T-bet is largely redundant for *in vivo* functions of human B cells and humoral immunity, it has a nuanced role in regulating Ig CSR, evidenced by increased serum levels of IgG1, IgG4 and IgE, reduced serum IgG2 levels, and increased proportions of IgG1^+^ and IgG4^+^ memory B cells, in a patient with complete T-bet deficiency. The alteration to IgG subclasses and serology is unlikely to reflect infectious history of the patient as he has been in remission and free of mycobacterial infection for several years. These perturbations to Ig levels are likely results from B-cell intrinsic and/or extrinsic mechanisms. On one hand, T-bet may directly regulate IgG subclasses in human B cells. On the other hand, increased serum IgG1, IgG4 and IgE in T-bet deficiency are consistent with skewing of T-bet deficient CD4^+^ T cells to a T_H_2-type effector function, evidenced by increased production of IL-4, IL-5 and IL-13 (56), and the well-established role of these cytokines in inducing human B-cell class switching to IgG1, IgG4 and IgE (59). Thus, dysregulated T_H_2 cytokine production by T-bet deficient CD4^+^ T cells may contribute to altered levels of some serum Ig classes in the patient.

These findings from human T-bet deficiency are similar to those from mice which established that T-bet is required for CSR to IgG2a/c *in vitro* and *in vivo* (10, 11, 15, 16). Interestingly, B-cell intrinsic T-bet-dependent IgG2a/c production appears to be important in mice *in vivo* for long-lived humoral immunity following immunization (16) or during viral and parasitic infections (17-20), and in the pathogenesis of autoimmune disease models (30, 45, 91). Despite altered serum Ig levels in the human T-bet deficient patient, he has not presented any clinical disease due to infections with, for example, *S. pneumoniae*, to which he has been exposed and can be life-threatening in patients with B-cell immunodeficiency disorders (92, 93). This is also consistent with our findings of intact SHM, affinity maturation, memory B-cell formation in the patient, as well as intact differentiation of his naïve and memory B cells into Ig-secreting cells in response to polyclonal stimulation *in vitro*. Thus, these clinical and immunological features suggest T-bet constrains CSR to IgG1, IgG2, and IgG4, but is largely redundant for clinically meaningful B cell-mediated humoral immunity against most common infections in humans, at least for the functions tested to date.

Whilst humoral immunity was essentially unaffected by T-bet deficiency, a major discovery from our study was that T-bet is essential for the generation of the CD11c^hi^CXCR3^+^ subset of human CD21^lo^CD19^hi^ B cells *in vivo* and *in vitro*. We also identified pathways upstream of T-bet fundamental for generating human CD11c^hi^CXCR3^+^CD21^lo^CD19^hi^ B cells. *In vitro* co-stimulation of human naïve B cells with TLR9, BCR, and either IFN-γ or IL-27 induced high level co-expression of T-bet, CXCR3, FCRL5 and CD19. Despite this, neither IFN-γ nor IL-27 were uniquely required to generate human T-bet^+^ B cells *in vivo*, as frequencies of these cells were intact in patients with IFN-γR or IL-27R-deficiencies. However, CD21^lo^CD11c^hi^CD19^hi^CD20^hi^CXCX3^+^ B cells were reduced in peripheral blood of patients with complete AR STAT1 deficiency, which impairs IFN-γ and IL-27 signaling, or IFN-γR1 deficiency, which abolishes IFN-γ signaling. These findings indicate that T-bet and STAT1, downstream of IFN-γ or IL-27, co-operate to induce the transcriptomic and epigenetic imprinting necessary to generate CD21^lo^CD11c^+^ B cells *in vivo*. Furthermore, these cytokines compensate for each another in individuals with defective signaling due to loss-of-function mutations in *IFNGR1* or *IL27R*. As CD21^lo^T-bet^+^ B cells are overrepresented in several human immune dysregulatory diseases, these findings indicate that a selective JAK1/STAT1 inhibitor, or directly targeting T-bet, may yield beneficial clinical outcomes by preventing or controlling expansion of pathogenic CD21^lo^T-bet^+^ B cells. Indeed, JAK inhibitors can suppress the *in vitro* generation of T-bet^+^ B cells from naïve B cells from healthy donors (67).

The physiological or pathogenic roles of CD21^lo^CD11c^+^ B cells remain enigmatic. First, although frequencies of CD21^lo^CD11c^+^ B cells increases in peripheral blood following vaccination, chronic infections and in autoimmune disorders (9, 31, 47, 50-52, 39-46), the predominance of these cells in these conditions is largely correlative. Furthermore, it remains unclear how they contribute to immunopathology. Similarly, it is unclear whether the expansion of these cells is a cause or consequence of the immune stimulatory environment of infection or autoimmunity. Second, despite lacking CD21^lo^CD11c^+^ B cells, the T-bet deficient patient has largely normal humoral immunity *in vivo* and B-cell function *in vitro,* despite skewing of IgG subclasses. It is thus possible that CD21^lo^CD11c^+^ B cells play roles in processes other than humoral immunity. Indeed, spectral flow cytometry and CITE-seq revealed an enrichment in expression of genes encoding proteins involved in antigen presentation, cognate T-B cell interactions, and peripheral tolerance in CD21^lo^CD11c^+^ B cells. As the T-bet-deficient patient is young, long-term follow up may reveal whether he is protected from or prone to certain conditions. The identification of additional T-bet-deficient patients is required to draw firm conclusions. Overall, findings from our study establish a framework to investigate CD21^lo^CD11c^+^ B cells in human health and disease, particularly other patients with known or newly discovered genetic defects. These future studies will shed more light on the molecular requirements for the development and function of this intriguing B-cell subset.

## MATERIALS AND METHODS

### Study design

We investigated the B cell and antibody phenotypes in a patient with autosomal recessive T-bet deficiency. We also enrolled his relatives and healthy controls in the study as controls. We performed *ex vivo* and *in vitro* experiments using peripheral blood mononuclear cells derived from the patient and controls. We also obtained DNA, plasma, and other biospecimens from the patient and controls to analyze their *in vivo* phenotypes. Both biological and technical replicates were used to validate the findings. Experiments were performed at least twice with appropriate replications. Conclusions were drawn from analyzing the results from aforementioned approaches collectively.

### Human subjects

The T-bet-deficient patient and the relatives studied here were living in and followed up in Morocco. The case report has already been published (55, 56). The study was approved by and performed in accordance with the requirements of the institutional ethics committees of Necker Hospital for Sick Children, Paris, France, and the Rockefeller University, New York, USA. Informed consent was obtained from the patient, his relatives, and the healthy control volunteers enrolled in the study. This study was also approved by the Sydney Local Health District RPAH Zone Human Research Ethics Committee and Research Governance Office, Royal Prince Alfred Hospital, Camperdown, New South Wales, Australia (protocol X16-0210/LNR/16/RPAH/257). Experiments using samples from human subjects were conducted in the United States, France, Australia and Sweden, in accordance with local regulations and with the approval of the IRBs of corresponding institutions.

### Bulk sequencing and analysis of immunoglobulin transcripts from PBMCs

Immunoglobulin heavy chain (*IGH*), kappa chain (*IGK*) and lambda chain (*IGL*) repertoires were sequenced from PBMCs from the T-bet-deficient patient (4 years of age at the time of sampling) and five healthy donors aged 11 months, 24 months, 16 years, 45 years and 66 years. Independent amplifications for each isotype (IgM, IgG, IgA and IgE) were performed for the heavy chain, and IGK and IGL were amplified in separate reactions, as previously described, with the addition of IgA and IgE reverse primers (94); IgA: GTCTCGTGGGCTCGGAGATGTGTATAAGAGACAGCAGGTCACACTGAGTGGCTCC, IgE: GTCTCGTGGGCTCGGAGATGTGTATAAGAGACAGCCAGGCAGCCCAGAGTCACGG. Samples were indexed and pooled for sequencing on an Illumina NextSeq with 2x300 PE.

Sample datasets were demultiplexed during FASTQ generation on the basis of sample indices. Paired-end reads were merged with FLASH (95) and the merged sequences were quality-filtered with FilterSeq from the presto (v0.5.13 2019.08.29) package (96), with a minimum quality of 20. Forward and reverse primers were trimmed, and constant regions were tagged with MaskPrimers (presto package), with a requirement for exact matches and the discarding of reads not meeting this requirement. Datasets were deduplicated (only unique sequences retained) with CollapseSeq (presto package) and the deduplicated datasets were input into stand-alone IgBLAST (v1.14) (97) for alignment against the IMGT human germline reference directories (downloaded 16 Jan 2020). IgBLAST results were filtered to remove truncated transcripts and transcripts lacking an identifiable CDR3. B-cell clones were inferred for each subject for IGH (combining transcripts from all isotypes), IGK and IGL. Clones were generated by first subsetting the VDJs from each donor on the basis of V gene, J gene and CDR3 length and then clustering CDR3 nucleotide sequences, with a 90% threshold, with cd-hit (98). Each cluster was inferred to be a clone of related VDJs stemming from a lineage that shared the same progenitor B cell. Median somatic hypermutation (SHM) for each clone was calculated per isotype for the V-REGION (percentage of V-REGION nucleotides mutated, based on IgBLAST alignment), and clone size, as both total and unique read numbers were also calculated.

### B-cell differentiation

Naïve (CD20^+^CD10^−^CD27^−^IgG^−^) and memory (CD20^+^CD10^−^CD27^+^IgG^+^) B cells were purified by sorting from the PBMCs of healthy donors or P with a FACSAria III. Purity was typically >97%. We assessed the *in vitro* induction of T-bet^+^ B cells by culturing naïve B cells in media alone (RPMI1640/10% FCS), or with presence of F(ab’)_2_ goat anti-human Ig (0.8 μg/mL) and CpG (0.35 μg/mL) with or without IFN-γ (333 U/mL) or IL-27 (50 ng/mL). After 3.5 days, the B cells were harvested, and stained for the surface expression of CD19, FCRL5 and CXCR3, fixed and permeabilized and then stained for intracellular expression of T-bet. Proportions of T-bet^+^ B cells, and expression of CD19, FCRL5 and CXCR3 on T-bet^+^ and T-bet^−^ B cells present in the cultures, were then determined. B-cell viability was determined with the Zombie Aqua Viability dye (BioLegend). We investigated *in vitro* differentiation into Ig-secreting cells, by culturing naïve and memory B cells with CD40L (200 ng/mL) cross-linked with anti-HA mAb (50 ng/mL, R&D Systems) alone or together with IL-21 (50 ng/mL, PeproTech), IL-10 (100 U/mL; provided by R. de Waal Malefyt - DNAX Research Institute, Palo Alto, CA), IL-21 plus IL-4 (100 U/mL; provided by R. de Waal Malefyt), or CpG 2006 (1 μg/mL, Sigma-Aldrich). Culture supernatants were harvested after 7 days and the amount of IgM, IgG and IgA secreted into the supernatant was determined in Ig heavy chain-specific ELISAs (61, 99). Secretion of IgG1, IgG2, IgG3, and IgG4 was determined using an IgG subclass ELISA kit (Invitrogen, catalogue # 99-1000) as per manufacturers’ instructions.

### Immunophenotyping of age-associated B cells with spectral flow cytometry

Experiments were performed in two batches. In the first batch, PBMCs were obtained from 20 healthy adult donors, four age-matched controls (2, 6, 7, 8 years of age), P (4 years of age at the time of sampling), P's healthy brother (8 years of age at the time of sampling), who is wild-type for the *TBX21* locus, and P’s healthy mother, who is heterozygous for the mutation (55, 100-102). In the second batch, PBMCs were obtained from 10 healthy adult donors and a patient with complete STAT1 deficiency (68). We stained 1 x 10^6^ to 2 x 10^6^ PBMCs from each individual with Zombie-NIR live-dead exclusion dye (BioLegend). Cells were then labeled with FcBlock (Miltenyi Biotec), and then with antibodies (Abs) against surface antigens, including anti-CD10-BUV737 (BD Biosciences), anti-CD23-BUV805 (BD Biosciences), anti-CD80-FITC (BioLegend), anti-FcRL4-PERCP/Cy5.5 (BioLegend), anti-CD138-PE/Dazzle594 (BioLegend), anti-FcRL5-APC (BioLegend), anti-CD269-APC/Fire750 (BioLegend), anti-CXCR3-BUV496 (BD Biosciences), anti-CD20-Alexa532 (Thermo Fisher Scientific), anti-CD11b-BUV395 (BD Biosciences), anti-CD38-BUV661 (BD Biosciences), anti-CD24-BUV563 (BD Biosciences), anti-CD56-V450 (BD Biosciences), anti-CD3-V450 (BD Biosciences), anti-CD5-BV480 (BD Biosciences), anti-IgM-BV570 (BioLegend), anti-CD95-BV605 (BioLegend), anti-HLA-DR-Qdot605 (Thermo Fisher Scientific), anti-CD40-BV650 (BioLegend), anti-IgD-BV785 (BioLegend), anti-CD21-PE (BD Biosciences), anti-CD86-Alexa647 (BioLegend), anti-CD11c-Alexa700 (BioLegend), anti-CD27-BV711 (BioLegend), anti-CCR7-BV750 (BioLegend), anti-CXCR4-BV421 (BioLegend), anti-CD19-Spark/NIR685 (BioLegend), and anti-IgG-PE/Cy5 (BD Biosciences) antibodies. The cells were then fixed and permeabilized with the FOXP3/Transcription factor staining buffer set (eBioscience). Cells were subjected to intracellular staining with anti-T-bet-PE/Cy7 antibody overnight (BioLegend). Data were acquired by spectral flow cytometry (Cytek). It should be noted that IgG-PE/Cy5 signal was found to be masked by a humanized FcBlock antibody which was mistakenly used in the study. Due to this technical reason, IgG signal was almost undetectable from this immunophenotyping. However, IgG-PE/Cy5 did not affect the balance of other 28 markers in this spectral flow experiment.

### Cellular indexing of transcriptomes and epitopes by sequencing (CITE-seq)

PBMCs from four age-matched controls (sample codes: #906, #4156, #1093, and #14881 with ages ranging from 2 to 8 years), three IFN-γR1-deficient patients (sample codes: #1114, #13391, #15553), the T-bet-deficient patient (P or M/M – referring to genotype Mutant/Mutant), and P's healthy brother (WT/WT) were isolated and frozen on different dates (55, 100-102). We stained 3 x 10^6^ PBMCs from each individual with Zombie-NIR live-dead exclusion dye (BioLegend). Cells were labeled with FcBlock (Miltenyi Biotech), and then with an antibody pool containing 1 μg of each BioLegend TotalSeq™ Ab, including anti-CD21 (clone Bu32), anti-CD11c (clone S-HCL-3), anti-FcRL5 (clone 509f6), anti-CXCR3 (clone G025H7), anti-CD95 Abs (clone DX2). Cells from these nine individuals were labeled individually with TotalSeq™ anti-human Hashtag Abs, including TotalSeq™-C0251 (age-matched control 906), C0252 (age-matched control 4156), C0253 (age-matched control 1093), C0254 (age-matched control 14881), C0255 (IFN-γR1-deficient patient 1114), C0256 (P's healthy brother 15645), C0257 (IFN-γR1-deficient patient 13391), C0258 (IFN-γR1-deficient patient 15553), and C0259 (P). Cells were washed twice with 2% FBS in PBS, pooled together, and subjected to staining with FcBlock (Miltenyi Biotech), anti-CD27-BV711 (BioLegend), anti-CD10-APC (BioLegend), anti-CD56-V450 (BD Biosciences), anti-CD3-V450 (BD Biosciences), anti-CD20-FITC (BioLegend), anti-CD21-PE (BD Biosciences Clone B-Ly4), and anti-CD11c-Alexa700 (BioLegend Clone S-HCL-3) Abs. The pooled cells were washed once and subjected to FACS. Live CD3^−^CD56^−^CD20^+^CD21^lo^ B cells were sorted and subjected to single-cell RNA-seq with the 10 x Genomics platform. It should be noted that anti-CD11c TotalSeq Ab and CD11c-Alexa700 did not compete against each other. CD21-PE FACS Ab and anti-CD21-TotalSeq Ab did not compete against each other either.

Single-cell 5' expression and BCR libraries were generated with the Chromium Single Cell 5’ Library & Gel Bead kit Version 2 (10x Genomics cat # PN1000265). BCR cDNA was amplified from the total cDNA pool with the 10X Chromium Single-Cell Human BCR Amplification Kit (PN-1000253) before library construction. Hashtag libraries were generated with the Chromium Single Cell 5' Feature Barcode Kit (PN 1000256). Standard protocols from 10X Genomics were followed for library generation. The quality of the libraries was assessed on an Agilent TapeStation and the three libraries were pooled in the following ratio: expression 10:BCR 1:HTO 1. The pooled libraries were sequenced on an Illumina NovaSeq 6000 sequencer with a 100-cycle SP flow cell. In total, 800 million paired reads were generated (read 1 = 26 bp, read 2 = 90 bp).

### Additional materials and methods located in Supplemental Methods

Some detailed materials and methods are provided in a Supplemental Methods section. This material includes the methods used for analysis of B cells with conventional flow cytometry, immunophenotyping of surface B cell receptors with spectral flow cytometry and unsupervised analysis of data from spectral flow cytometry. The Supplemental Methods section also describes the methods used for real-time quantitative *ENC1* PCR, analysis of CITE-seq data, single-cell VDJ sequencing analysis, naïve B-cell differentiation for Omni-ATAC-seq, and analysis of Omni-ATAC-seq.

### Statistical analysis

Student’s t-test, Mann-Whitney test, one-way ANOVA, and two-way ANOVA were used in their corresponding datasets to investigate statistical difference. Bar graphs throughout the figures represent either the mean and the standard deviation or the mean and the standard error of the mean. Dots present individual samples or technical replicates. P values of 0.05 and below are considered to be statistically significant. *p<0.05, **p<0.01, ***p<0.001, ****p<0.0001, and ns = not significant (or not marked). Details of the statistical methods used in individual experiments are provided in the corresponding figure captions. All raw data are provided in Data file S4.

## Supplementary Material

data file S3 Data file S3. List of Cite-seq and ATAC-seq samples.

data file S4 Data file S4. Raw data file (Excel spreadsheet)

data file S2 Data file S2. ATAC-seq of CD21^lo^ B cells.

reproducibility checklist Data file. Reproducibility checklist.

main supplementary Figure S1. Polyclonal and antigen-specific antibody responses in inherited T-bet deficiency.
Figure S2. Characterization of the CD21^lo^CD11c^+^ B-cell subset.
Figure S3. Unsupervised FlowSOM analysis of B cells from a patient with inherited human T-bet deficiency.
Figure S4. Enrichment of IgG- or IgA-expressing B cells in CD21^lo^CD11c^+^ B cells.
Figure S5. Proteotranscriptomic investigation of CD21^lo^ B cells from a patient with inherited human T-bet deficiency.
Figure S6. Chromatin accessibilities of B cells are altered in inherited T-bet deficiency.
Table S1. Vaccine-specific antibody responses in T-bet deficiency. Plasma samples prepared when the patient was 6-month-, 2-year-, and 3-year-old were measured for the levels of antigen-specific antibodies. Units are IU/mL if not otherwise specified. ND not detectable.

data file S1 Data file S1. CITE-seq of CD21^lo^ B cells.

## References

[1] Szabo SJ, Kim ST, Costa GL, Zhang X, Fathman CG, Glimcher LH, A novel transcription factor, T-bet, directs Th1 lineage commitment. Cell. 100, 655–669 (2000). 10761931 10.1016/s0092-8674(00)80702-3

[2] Szabo SJ, Sullivan BM, Stemmann C, Satoskar AR, Sleckman BP, Glimcher LH, Distinct effects of T-bet in TH1 lineage commitment and IFN-gamma production in CD4 and CD8 T cells. Science (80-. ). 295, 338–342 (2002). 10.1126/science.1065543 11786644

[3] Lazarevic V, Glimcher LH, Lord GM, T-bet: a bridge between innate and adaptive immunity. Nat Rev Immunol. 13, 777–789 (2013). 24113868 10.1038/nri3536 PMC6290922

[4] Hao Y, O’Neill P, Naradikian MS, Scholz JL, Cancro MP, A B-cell subset uniquely responsive to innate stimuli accumulates in aged mice. Blood. 118, 1294–1304 (2011). 21562046 10.1182/blood-2011-01-330530 PMC3152496

[5] Rubtsov AV, Rubtsova K, Fischer A, Meehan RT, Gillis JZ, Kappler JW, Marrack P, Toll-like receptor 7 (TLR7)-driven accumulation of a novel CD11c+ B-cell population is important for the development of autoimmunity. Blood (2011), doi:10.1182/blood-2011-01-331462. PMC3152497 21543762

[6] Rubtsova K, Rubtsov AV, Van Dyk LF, Kappler JW, Marrack P, T-box transcription factor T-bet, a key player in a unique type of B-cell activation essential for effective viral clearance. Proc. Natl. Acad. Sci. U. S. A 110, E3216 (2013). 23922396 10.1073/pnas.1312348110 PMC3752276

[7] Naradikian MS, Myles A, Beiting DP, Roberts KJ, Dawson L, Herati RS, Bengsch B, Linderman SL, Stelekati E, Spolski R, Wherry EJ, Hunter C, Hensley SE, Leonard WJ, Cancro MP, Cutting Edge: IL-4, IL-21, and IFN-γ Interact To Govern T-bet and CD11c Expression in TLR-Activated B Cells. J. Immunol 197, 1023–1028 (2016). 27430719 10.4049/jimmunol.1600522 PMC4975960

[8] Myles A, Gearhart PJ, Cancro MP, Signals that drive T-bet expression in B cells. Cell. Immunol (2017), doi:10.1016/j.cellimm.2017.09.004. PMC6191851 28923237

[9] Cancro MP, Age-Associated B Cells. Annu. Rev. Immunol 38, 315–340 (2020). 31986068 10.1146/annurev-immunol-092419-031130

[10] Gerth AJ, Lin L, Peng SL, T-bet regulates T-independent IgG2a class switching. Int Immunol. 15, 937–944 (2003). 12882831 10.1093/intimm/dxg093

[11] Liu N, Ohnishi N, Ni L., Akira S, Bacon KB, CpG directly induces T-bet expression and inhibits IgG1 and IgE switching in B cells. Nat. Immunol 4, 687–693 (2003). 12766768 10.1038/ni941

[12] Xu W, Zhang JJ, Stat1-Dependent Synergistic Activation of T-bet for IgG2a Production during Early Stage of B Cell Activation. J. Immunol 175, 7419–7424 (2005). 16301649 10.4049/jimmunol.175.11.7419

[13] Kövesdi D, Angyal A, Huber K, Szili D, Sármay G, T-bet is a new synergistic meeting point for the BCR and TLR9 signaling cascades. Eur. J. Immunol 44, 887–893 (2014). 24249581 10.1002/eji.201343841

[14] Yoshimoto T, Okada K, Morishima N, Kamiya S, Owaki T, Asakawa M, Iwakura Y, Fukai F, Mizuguchi J, Induction of IgG2a Class Switching in B Cells by IL-27. J. Immunol 173, 2479–2485 (2004). 15294962 10.4049/jimmunol.173.4.2479

[15] Peng SL, Szabo SJ, Glimcher LH, T-bet regulates IgG class switching and pathogenic autoantibody production. Proc. Natl. Acad. Sci. U. S. A 99, 5545–5550 (2002). 11960012 10.1073/pnas.082114899 PMC122806

[16] Wang NS, McHeyzer-Williams LJ, Okitsu SL, Burris TP, Reiner SL, McHeyzer-Williams MG, Divergent transcriptional programming of class-specific B cell memory by T-bet and RORα. Nat. Immunol 13, 604–611 (2012). 22561605 10.1038/ni.2294 PMC3362691

[17] Stone SL, Peel JN, Scharer CD, Risley CA, Chisolm DA, Schultz MD, Yu B, Ballesteros-Tato A, Wojciechowski W, Mousseau B, Misra RS, Hanidu A, Jiang H, Qi Z, Boss JM, Randall TD, Brodeur SR, Goldrath AW, Weinmann AS, Rosenberg AF, Lund FE, T-bet Transcription Factor Promotes Antibody-Secreting Cell Differentiation by Limiting the Inflammatory Effects of IFN-γ on B Cells. Immunity (2019), doi:10.1016/j.immuni.2019.04.004. PMC6929688 31076359

[18] Barnett BE, Staupe RP, Odorizzi PM, Palko O, Tomov VT, Mahan AE, Gunn B, Chen D, Paley MA, Alter G, Reiner SL, Lauer GM, Teijaro JR, Wherry EJ, Cutting Edge: B Cell–Intrinsic T-bet Expression Is Required To Control Chronic Viral Infection. J. Immunol 197, 1017–1022 (2016). 27430722 10.4049/jimmunol.1500368 PMC4975981

[19] Ly A, Liao Y, Pietrzak H, Ioannidis LJ, Sidwell T, Gloury R, Doerflinger M, Triglia T, Qin RZ, Groom JR, Belz GT, Good-Jacobson KL, Shi W, Kallies A, Hansen DS, Transcription Factor T-bet in B Cells Modulates Germinal Center Polarization and Antibody Affinity Maturation in Response to Malaria. Cell Rep. 29, 2257–2269.e6 (2019). 31747599 10.1016/j.celrep.2019.10.087

[20] Sheikh AA, Cooper L, Feng M, Souza-Fonseca-Guimaraes F, Lafouresse F, Duckworth BC, Huntington ND, Moon JJ, Pellegrini M, Nutt SL, Belz GT, Good-Jacobson KL, Groom JR, Context-Dependent Role for T-bet in T Follicular Helper Differentiation and Germinal Center Function following Viral Infection. Cell Rep. 28, 1758–1772.e4 (2019). 31412245 10.1016/j.celrep.2019.07.034 PMC6711398

[21] Mendoza A, Yewdell WT, Hoyos B, Schizas M, Bou-Puerto R, Michaels AJ, Brown CC, Chaudhuri J, Rudensky AY, Assembly of a spatial circuit of T-bet–expressing T and B lymphocytes is required for antiviral humoral immunity. Sci. Immunol 6, eabi4710 (2021). 34117110 10.1126/sciimmunol.abi4710 PMC8418793

[22] Johnson JL, Rosenthal RL, Knox JJ, Myles A, Naradikian MS, Madej J, Kostiv M, Rosenfeld AM, Meng W, Christensen SR, Hensley SE, Yewdell J, Canaday DH, Zhu J, McDermott AB, Dori Y, Itkin M, Wherry EJ, Pardi N, Weissman D, Naji A, Prak ETL, Betts MR, Cancro MP, The Transcription Factor T-bet Resolves Memory B Cell Subsets with Distinct Tissue Distributions and Antibody Specificities in Mice and Humans. Immunity. 52, 842–855.e6 (2020). 32353250 10.1016/j.immuni.2020.03.020 PMC7242168

[23] Rubtsov AV, Rubtsova K, Fischer A, Meehan RT, Gillis JZ, Kappler JW, Marrack P, Toll-like receptor 7 (TLR7)-driven accumulation of a novel CD11c+ B-cell population is important for the development of autoimmunity. Blood. 118, 1305–1315 (2011). 21543762 10.1182/blood-2011-01-331462 PMC3152497

[24] Ratliff M, Alter S, Frasca D, Blomberg BB, Riley RL, In senescence, age-associated b cells secrete tnfα and inhibit survival of b-cell precursors. Aging Cell. 12, 303–311 (2013). 23410004 10.1111/acel.12055 PMC3716274

[25] Russell Knode LM, Naradikian MS, Myles A, Scholz JL, Hao Y, Liu D, Ford ML, Tobias JW, Cancro MP, Gearhart PJ, Age-Associated B Cells Express a Diverse Repertoire of V H and Vκ Genes with Somatic Hypermutation. J. Immunol 198, 1921–1927 (2017). 28093524 10.4049/jimmunol.1601106 PMC5322232

[26] Racine R, Chatterjee M, Winslow GM, CD11c Expression Identifies a Population of Extrafollicular Antigen-Specific Splenic Plasmablasts Responsible for CD4 T-Independent Antibody Responses during Intracellular Bacterial Infection. J. Immunol 181, 1375–1385 (2008). 18606692 10.4049/jimmunol.181.2.1375 PMC2645789

[27] Papillion AM, Kenderes KJ, Yates JL, Winslow GM, Early derivation of IgM memory cells and bone marrow plasmablasts. PLoS One. 12, e0178853 (2017). 28575114 10.1371/journal.pone.0178853 PMC5456393

[28] Rivera-Correa J, Guthmiller JJ, Vijay R, Fernandez-Arias C, Pardo-Ruge MA, Gonzalez S, Butler NS, Rodriguez A, Plasmodium DNA-mediated TLR9 activation of T-bet+ B cells contributes to autoimmune anaemia during malaria. Nat. Commun 8 (2017), doi:10.1038/s41467-017-01476-6. PMC5670202 29101363

[29] Manni M, Gupta S, Ricker E, Chinenov Y, Park SH, Shi M, Pannellini T, Jessberger R, Ivashkiv LB, Pernis AB, Regulation of age-associated B cells by IRF5 in systemic autoimmunity. Nat. Immunol 19, 407–419 (2018). 29483597 10.1038/s41590-018-0056-8 PMC6095139

[30] Rubtsova K, Rubtsov AV, Thurman JM, Mennona JM, Kappler JW, Marrack P, B cells expressing the transcription factor T-bet drive lupus-like autoimmunity. J. Clin. Invest 127, 1392–1404 (2017). 28240602 10.1172/JCI91250 PMC5373868

[31] Liu Y, Zhou S, Qian J, Wang Y, Yu X, Dai D, Dai M, Wu L, Liao Z, Xue Z, Wang J, Hou G, Ma J, Harley JB, Tang Y, Shen N, T-bet+CD11c+ B cells are critical for antichromatin immunoglobulin G production in the development of lupus. Arthritis Res. Ther 19, 225 (2017). 28982388 10.1186/s13075-017-1438-2 PMC5629756

[32] Rubtsov AV, Rubtsova K, Kappler JW, Marrack P, TLR7 drives accumulation of ABCs and autoantibody production in autoimmune-prone mice. Immunol. Res 55, 210–216 (2013). 22945807 10.1007/s12026-012-8365-8 PMC3935605

[33] Du SW, Arkatkar T, Jacobs HM, Rawlings DJ, Jackson SW, Generation of functional murine CD11c + age-associated B cells in the absence of B cell T-bet expression. Eur. J. Immunol 49, 170–178 (2019). 30353919 10.1002/eji.201847641 PMC6779321

[34] Levack RC, Newell KL, Popescu M, Cabrera-Martinez B, Winslow GM, CD11c + T-bet + B Cells Require IL-21 and IFN-γ from Type 1 T Follicular Helper Cells and Intrinsic Bcl-6 Expression but Develop Normally in the Absence of T-bet. J. Immunol 205, 1050–1058 (2020). 32680956 10.4049/jimmunol.2000206 PMC7556359

[35] Warnatz K, Denz A, Dräger R, Braun M, Groth C, Wolff-Vorbeck G, Eibel H, Schlesier M, Peter HH, Severe deficiency of switched memory B cells (CD27+IgM-IgD-) in subgroups of patients with common variable immunodeficiency: A new approach to classify a heterogeneous disease. Blood. 99, 1544–1551 (2002). 11861266 10.1182/blood.v99.5.1544

[36] Weiss GE, Crompton PD, Li S, Walsh LA, Moir S, Traore B, Kayentao K, Ongoiba A, Doumbo OK, Pierce SK, Atypical Memory B Cells Are Greatly Expanded in Individuals Living in a Malaria-Endemic Area. J. Immunol 183, 2176–2182 (2009). 19592645 10.4049/jimmunol.0901297 PMC2713793

[37] Portugal S, Tipton CM, Sohn H, Kone Y, Wang J, Li S, Skinner J, Virtaneva K, Sturdevant DE, Porcella SF, Doumbo OK, Doumbo S, Kayentao K, Ongoiba A, Traore B, Sanz I, Pierce SK, Crompton PD, Malaria-associated atypical memory B cells exhibit markedly reduced B cell receptor signaling and effector function. Elife. 4 (2015), doi:10.7554/eLife.07218. PMC4444601 25955968

[38] Knox JJ, Kaplan DE, Betts MR, T-bet-expressing B cells during HIV and HCV infections. Cell. Immunol 321, 26–34 (2017). 28739077 10.1016/j.cellimm.2017.04.012 PMC5732046

[39] Moir S, Ho J, Malaspina A, Wang W, DiPoto AC, O’Shea MA, Roby G, Kottilil S, Arthos J, Proschan MA, Chun TW, Fauci AS, Evidence for HIV-associated B cell exhaustion in a dysfunctional memory B cell compartment in HIV-infected viremic individuals. J. Exp. Med 205, 1797–1805 (2008). 18625747 10.1084/jem.20072683 PMC2525604

[40] Austin JW, Buckner CM, Kardava L, Wang W, Zhang X, Melson VA, Swanson RG, Martins AJ, Zhou JQ, Hoehn KB, Nicholas Fisk J, Dimopoulos Y, Chassiakos A, O’Dell S, Smelkinson MG, Seamon CA, Kwan RW, Sneller MC, Pittaluga S, Doria-Rose NA, McDermott A, Li Y, Chun TW, Kleinstein SH, Tsang JS, Petrovas C, Moir S, Overexpression of T-bet in HIV infection is associated with accumulation of B cells outside germinal centers and poor affinity maturation. Sci. Transl. Med 11 (2019), doi:10.1126/scitranslmed.aax0904. PMC7479651 31776286

[41] Chang LY, Li Y, Kaplan DE, Hepatitis C viraemia reversibly maintains subset of antigen-specific T-bet+ tissue-like memory B cells. J. Viral Hepat 24, 389–396 (2017). 27925349 10.1111/jvh.12659 PMC5637374

[42] Rivera-Correa J, Mackroth MS, Jacobs T, Zur Wiesch JS, Rolling T, Rodriguez A, Atypical memory b-cells are associated with plasmodium falciparum anemia through anti-phosphatidylserine antibodies. Elife. 8 (2019), doi:10.7554/eLife.48309. PMC6853636 31713516

[43] Wang S, Wang J, Kumar V, Karnell JL, Naiman B, Gross PS, Rahman S, Zerrouki K, Hanna R, Morehouse C, Holoweckyj N, Liu H, Casey K, Smith M, Parker M, White N, Riggs J, Ward B, Bhat G, Rajan B, Grady R, Groves C, Manna Z, Goldbach-Mansky R, Hasni S, Siegel R, Sanjuan M, Streicher K, Cancro MP, Kolbeck R, Ettinger R, IL-21 drives expansion and plasma cell differentiation of autoreactive CD11chiT-bet+ B cells in SLE. Nat. Commun 9 (2018), doi:10.1038/s41467-018-03750-7. PMC5931508 29717110

[44] Zumaquero E, Stone SL, Scharer CD, Jenks SA, Nellore A, Mousseau B, Vela AR, Botta D, Bradley JE, Wojciechowski W, Ptacek T, Danila MI, Edberg JC, Bridges SL, Kimberly RP, Chatham WW, Schoeb TR, Rosenberg AF, Boss JM, Sanz I, Lund FE, Ifnγ induces epigenetic programming of human t-bethi b cells and promotes tlr7/8 and il-21 induced differentiation. Elife. 8 (2019), doi:10.7554/eLife.41641. PMC6544433 31090539

[45] Jenks SA, Cashman KS, Zumaquero E, Marigorta UM, Patel AV, Wang X, Tomar D, Woodruff MC, Simon Z, Bugrovsky R, Blalock EL, Scharer CD, Tipton CM, Wei C, Lim SS, Petri M, Niewold TB, Anolik JH, Gibson G, Lee FEH, Boss JM, Lund FE, Sanz I, Distinct Effector B Cells Induced by Unregulated Toll-like Receptor 7 Contribute to Pathogenic Responses in Systemic Lupus Erythematosus. Immunity. 49, 725–739.e6 (2018). 30314758 10.1016/j.immuni.2018.08.015 PMC6217820

[46] van Langelaar J, Rijvers L, Janssen M, Wierenga-Wolf AF, Melief MJ, Siepman TA, de Vries HE, Unger PPA, van Ham SM, Hintzen RQ, van Luijn MM, Induction of brain-infiltrating T-bet–expressing B cells in multiple sclerosis. Ann. Neurol 86, 264–278 (2019). 31136008 10.1002/ana.25508 PMC6771938

[47] Rakhmanov M, Keller B, Gutenberger S, Foerster C, Hoenig M, Driessen G, Van Der Burg M, Van Dongen JJ, Wiech E, Visentini M, Quinti I, Prasse A, Voelxen N, Salzer U, Goldacker S, Fisch P, Eibel H, Schwarz K, Peter HH, Warnatz K, Circulating CD21low B cells in common variable immunodeficiency resemble tissue homing, innate-like B cells. Proc. Natl. Acad. Sci. U. S. A 106, 13451–13456 (2009). 19666505 10.1073/pnas.0901984106 PMC2726348

[48] Isnardi I, Ng YS, Menard L, Meyers G, Saadoun D, Srdanovic I, Samuels J, Berman J, Buckner JH, Cunningham-Rundles C, Meffre E, Complement receptor 2/CD21- human naive B cells contain mostly autoreactive unresponsive clones. Blood. 115, 5026–5036 (2010). 20231422 10.1182/blood-2009-09-243071 PMC3373152

[49] Freudenhammer M, Voll RE, Binder SC, Keller B, Warnatz K, Naive- and Memory-like CD21 low B Cell Subsets Share Core Phenotypic and Signaling Characteristics in Systemic Autoimmune Disorders. J. Immunol 205, 2016–2025 (2020). 32907998 10.4049/jimmunol.2000343

[50] Lau D, Lan LYL, Andrews SF, Henry C, Rojas KT, Neu KE, Huang M, Huang Y, DeKosky B, Palm AKE, Ippolito GC, Georgiou G, Wilson PC, Low CD21 expression defines a population of recent germinal center graduates primed for plasma cell differentiation. Sci. Immunol 2 (2017), doi:10.1126/sciimmunol.aai8153. PMC5896567 28783670

[51] Andrews SF, Chambers MJ, Schramm CA, Plyler J, Raab JE, Kanekiyo M, Gillespie RA, Ransier A, Darko S, Hu J, Chen X, Yassine HM, Boyington JC, Crank MC, Chen GL, Coates E, Mascola JR, Douek DC, Graham BS, Ledgerwood JE, McDermott AB, Activation Dynamics and Immunoglobulin Evolution of Pre-existing and Newly Generated Human Memory B cell Responses to Influenza Hemagglutinin. Immunity. 51, 398–410.e5 (2019). 31350180 10.1016/j.immuni.2019.06.024

[52] Oliviero B, Varchetta S, Mele D, Mantovani S, Cerino A, Perotti CG, Ludovisi S, Mondelli MU, Expansion of atypical memory B cells is a prominent feature of COVID-19. Cell. Mol. Immunol 17, 1101–1103 (2020). 32879471 10.1038/s41423-020-00542-2 PMC7463104

[53] Wildner NH, Ahmadi P, Schulte S, Brauneck F, Kohsar M, Lütgehetmann M, Beisel C, Addo MM, Haag F, Schulze zur Wiesch J, B cell analysis in SARS-CoV-2 versus malaria: Increased frequencies of plasmablasts and atypical memory B cells in COVID-19. J. Leukoc. Biol 109, 77–90 (2021). 33617048 10.1002/JLB.5COVA0620-370RR PMC10016889

[54] Keller B, Strohmeier V, Harder I, Unger S, Payne KJ, Andrieux G, Boerries M, Felixberger PT, Landry JJM, Nieters A, Rensing-Ehl A, Salzer U, Frede N, Usadel S, Elling R, Speckmann C, Hainmann I, Ralph E, Gilmour K, Wentink MWJ, van der Burg M, Kuehn HS, Rosenzweig SD, Kölsch U, von Bernuth H, Kaiser-Labusch P, Gothe F, Hambleton S, Vlagea AD, Garcia AG, Alsina L, Markelj G, Avcin T, Vasconcelos J, Guedes M, Ding JY, Ku CL, Shadur B, Avery DT, Venhoff N, Thiel J, Becker H, Erazo-Borrás L, Trujillo-Vargas CM, Franco JL, Fieschi C, Okada S, Gray PE, Uzel G, Casanova JL, Fliegauf M, Grimbacher B, Eibel H, Ehl S, Voll RE, Rizzi M, Stepensky P, Benes V, Ma CS, Bossen C, Tangye SG, Warnatz K, The expansion of human T-bet high CD21 low B cells is T cell dependent. Sci. Immunol 6 (2021), doi:10.1126/SCIIMMUNOL.ABH0891. 34623902

[55] Yang R, Mele F, Worley L, Langlais D, Rosain J, Benhsaien I, Elarabi H, Croft CA, Doisne J-M, Zhang P, Weisshaar M, Jarrossay D, Latorre D, Shen Y, Han J, Ogishi M, Gruber C, Markle J, Al Ali F, Rahman M, Khan T, Seeleuthner Y, Kerner G, Husquin LT, Maclsaac JL, Jeljeli M, Errami A, Ailal F, Kobor MS, Oleaga-Quintas C, Roynard M, Bourgey M, El Baghdadi J, Boisson-Dupuis S, Puel A, Batteux F, Rozenberg F, Marr N, Pan-Hammarström Q, Bogunovic D, Quintana-Murci L, Carroll T, Ma CS, Abel L, Bousfiha A, Di Santo JP, Glimcher LH, Gros P, Tangye SG, Sallusto F, Bustamante J, Casanova J-L, Human T-bet Governs Innate and Innate-like Adaptive IFN-γ Immunity against Mycobacteria. Cell (2020), doi:10.1016/j.cell.2020.10.046. PMC7770098 33296702

[56] Yang R, Weisshaar M, Mele F, Benhsaien I, Dorgham K, Han J, Croft CA, Notarbartolo S, Rosain J, Bastard P, Puel A, Fleckenstein B, Glimcher LH, Di Santo JP, Ma CS, Gorochov G, Bousfiha A, Abel L, Tangye SG, Casanova JL, Bustamante J, Sallusto F, High Th2 cytokine levels and upper airway inflammation in human inherited T-bet deficiency. J. Exp. Med 218 (2021), doi:10.1084/JEM.20202726/212431. PMC8225679 34160550

[57] Blanco E, Pérez-Andrés M, Arriba-Méndez S, Contreras-Sanfeliciano T, Criado I, Pelak O, Serra-Caetano A, Romero A, Puig N, Remesal A, Torres Canizales J, López-Granados E, Kalina T, Sousa AE, van Zelm M, van der Burg M, van Dongen JJM, Orfao A, Age-associated distribution of normal B-cell and plasma cell subsets in peripheral blood. J. Allergy Clin. Immunol 141, 2208–2219.e16 (2018). 29505809 10.1016/j.jaci.2018.02.017

[58] Morbach H, Eichhorn EM, Liese JG, Girschick HJ, Reference values for B cell subpopulations from infancy to adulthood. Clin. Exp. Immunol 162, 271–279 (2010). 20854328 10.1111/j.1365-2249.2010.04206.x PMC2996594

[59] Moens L, Tangye SG, Cytokine-Mediated Regulation of Plasma Cell Generation: IL-21 Takes Center Stage. Front. Immunol 5 (2014), doi:10.3389/FIMMU.2014.00065. PMC3927127 24600453

[60] Tangye SG, Ferguson A, Avery DT, Ma CS, Hodgkin PD, Isotype Switching by Human B Cells Is Division-Associated and Regulated by Cytokines. J. Immunol 169, 4298–4306 (2002). 12370361 10.4049/jimmunol.169.8.4298

[61] Bryant VL, Ma CS, Avery DT, Li Y, Good KL, Corcoran LM, de Waal Malefyt R, Tangye SG, Cytokine-Mediated Regulation of Human B Cell Differentiation into Ig-Secreting Cells: Predominant Role of IL-21 Produced by CXCR5 + T Follicular Helper Cells. J. Immunol 179, 8180–8190 (2007). 18056361 10.4049/jimmunol.179.12.8180

[62] Avery DT, Ma CS, Bryant VL, Santner-Nanan B, Nanan R, Wong M, Fulcher DA, Cook MC, Tangye SG, STAT3 is required for IL-21 induced secretion of IgE from human naive B cells. Blood. 112, 1784–1793 (2008). 18579794 10.1182/blood-2008-02-142745

[63] Phalke S, Marrack P, Age (autoimmunity) associated B cells (ABCs) and their relatives. Curr. Opin. Immunol 55 (2018), pp. 75–80. 30388513 10.1016/j.coi.2018.09.007

[64] Naradikian MS, Hao Y, Cancro MP, Age-associated B cells: Key mediators of both protective and autoreactive humoral responses. Immunol. Rev 269, 118–129 (2016). 26683149 10.1111/imr.12380

[65] Wang S, Wang J, Kumar V, Karnell JL, Naiman B, Gross PS, Rahman S, Zerrouki K, Hanna R, Morehouse C, Holoweckyj N, Liu H, Casey K, Smith M, Parker M, White N, Riggs J, Ward B, Bhat G, Rajan B, Grady R, Groves C, Manna Z, Goldbach-Mansky R, Hasni S, Siegel R, Sanjuan M, Streicher K, Cancro MP, Kolbeck R, Ettinger R, IL-21 drives expansion and plasma cell differentiation of autoreactive CD11chiT-bet+ B cells in SLE. Nat. Commun 9 (2018), doi:10.1038/s41467-018-03750-7. PMC5931508 29717110

[66] Golinski M-L, Demeules M, Derambure C, Riou G, Maho-Vaillant M, Boyer O, Joly P, Calbo S, CD11c+ B Cells Are Mainly Memory Cells, Precursors of Antibody Secreting Cells in Healthy Donors. Front. Immunol 11, 32 (2020). 32158442 10.3389/fimmu.2020.00032 PMC7051942

[67] Keller B, Strohmeier V, Harder I, Unger S, Payne KJ, Andrieux G, Boerries M, Felixberger PT, Landry JJM, Nieters A, Rensing-Ehl A, Salzer U, Frede N, Usadel S, Elling R, Speckmann C, Hainmann I, Ralph E, Gilmour K, Wentink MWJ, van der Burg M, Kuehn HS, Rosenzweig SD, Kölsch U, von Bernuth H, Kaiser-Labusch P, Gothe F, Hambleton S, Vlagea AD, Garcia AG, Alsina L, Markelj G, Avcin T, Vasconcelos J, Guedes M, Ding JY, Ku CL, Shadur B, Avery DT, Venhoff N, Thiel J, Becker H, Erazo-Borrás L, Trujillo-Vargas CM, Franco JL, Fieschi C, Okada S, Gray PE, Uzel G, Casanova JL, Fliegauf M, Grimbacher B, Eibel H, Ehl S, Voll RE, Rizzi M, Stepensky P, Benes V, Ma CS, Bossen C, Tangye SG, Warnatz K, The expansion of human T-bethighCD21low B cells is T cell dependent. Sci. Immunol 6, 52 (2021). 10.1126/sciimmunol.abh0891 34623902

[68] Le Voyer T, Sonoko S, Tsumura M, Khan T, Esteve-Sole A, Al Saud BK, Gungor HE, ......., Bustamante J, Genetic, Immunological, and Clinical Features of 32 patients with Autosomal recessive STAT1 deficiency. J Immunol. In press (2021). 10.4049/jimmunol.2001451 PMC8702442 34183371

[69] Ogishi M, Yang R, Gruber C, Zhang P, Pelham SJ, Spaan AN, Rosain J, Chbihi M, Han JE, Rao VK, Kainulainen L, Bustamante J, Boisson B, Bogunovic D, Boisson-Dupuis S, Casanova J-L, Multibatch Cytometry Data Integration for Optimal Immunophenotyping. J. Immunol, ji2000854 (2020). 10.4049/jimmunol.2000854 PMC7855665 33229441

[70] Van Gassen S, Callebaut B, Van Helden MJ, Lambrecht BN, Demeester P, Dhaene T, Saeys Y, FlowSOM: Using self-organizing maps for visualization and interpretation of cytometry data. Cytom. Part A 87, 636–645 (2015). 10.1002/cyto.a.22625 25573116

[71] Ellebedy AH, Jackson KJL, Kissick HT, Nakaya HI, Davis CW, Roskin KM, McElroy AK, Oshansky CM, Elbein R, Thomas S, Lyon GM, Spiropoulou CF, Mehta AK, Thomas PG, Boyd SD, Ahmed R, Defining antigen-specific plasmablast and memory B cell subsets in blood following viral infection and vaccination of humans. Nat. Immunol 17, 1226 (2016). 27525369 10.1038/ni.3533 PMC5054979

[72] Reincke ME, Payne KJ, Harder I, Strohmeier V, Voll RE, Warnatz K, Keller B, The Antigen Presenting Potential of CD21low B Cells. Front. Immunol 11, 2664 (2020). 10.3389/fimmu.2020.535784 PMC7609862 33193306

[73] Tan C, Hiwa R, Mueller JL, Vykunta V, Hibiya K, Noviski M, Huizar J, Brooks JF, Garcia J, Heyn C, Li Z, Marson A, Zikherman J, NR4A nuclear receptors restrain B cell responses to antigen when second signals are absent or limiting. Nat. Immunol 21, 1267–1279 (2020). 32868928 10.1038/s41590-020-0765-7 PMC8081071

[74] Shi Z, Zhang Q, Yan H, Yang Y, Wang P, Zhang Y, Deng Z, Yu M, Zhou W, Wang Q, Yang X, Mo X, Zhang C, Huang J, Dai H, Sun B, Zhao Y, Zhang L, Yang YG, Qiu X, More than one antibody of individual B cells revealed by single-cell immune profiling. Cell Discov. 5, 1–13 (2019). 31839985 10.1038/s41421-019-0137-3 PMC6901605

[75] Brady BL, Steinel NC, Bassing CH, Antigen Receptor Allelic Exclusion: An Update and Reappraisal. J. Immunol 185, 3801–3808 (2010). 20858891 10.4049/jimmunol.1001158 PMC3008371

[76] Giudicelli V, Brochet X, Lefranc MP, IMGT/V-QUEST: IMGT standardized analysis of the immunoglobulin (IG) and T cell receptor (TR) nucleotide sequences. Cold Spring Harb. Protoc 6, 695–715 (2011). 10.1101/pdb.prot5633 21632778

[77] Brochet X, Lefranc MP, Giudicelli V, IMGT/V-QUEST: the highly customized and integrated system for IG and TR standardized V-J and V-D-J sequence analysis. Nucleic Acids Res. 36 (2008), doi:10.1093/nar/gkn316. PMC2447746 18503082

[78] Ambegaonkar AA, Nagata S, Pierce SK, Sohn H, The differentiation in vitro of human tonsil B cells with the phenotypic and functional characteristics of T-bet+ atypical memory B cells in malaria. Front. Immunol 10 (2019), doi:10.3389/fimmu.2019.00852. PMC6491666 31068937

[79] Bax HI, Freeman AF, Ding L, Hsu AP, Marciano B, Kristosturyan E, Jancel T, Spalding C, Pechacek J, Olivier KN, Barnhart LA, Boris L, Frein C, Claypool RJ, Anderson V, Zerbe CS, Holland SM, Sampaio EP, Interferon alpha treatment of patients with impaired interferon gamma signaling. J. Clin. Immunol 33, 991–1001 (2013). 23512243 10.1007/s10875-013-9882-5 PMC4136390

[80] Ma CS, Wong N, Rao G, Avery DT, Torpy J, Hambridge T, Bustamante J, Okada S, Stoddard JL, Deenick EK, Pelham SJ, Payne K, Boisson-Dupuis S, Puel A, Kobayashi M, Arkwright PD, Kilic SS, El Baghdadi J, Nonoyama S, Minegishi Y, Mahdaviani SA, Mansouri D, Bousfiha A, Blincoe AK, French MA, Hsu P, Campbell DE, Stormon MO, Wong M, Adelstein S, Smart JM, Fulcher DA, Cook MC, Phan TG, Stepensky P, Boztug K, Kansu A, Ikincioʇullari A, Baumann U, Beier R, Roscioli T, Ziegler JB, Gray P, Picard C, Grimbacher B, Warnatz K, Holland SM, Casanova JL, Uzel G, Tangye SG, Monogenic mutations differentially affect the quantity and quality of T follicular helper cells in patients with human primary immunodeficiencies. J. Allergy Clin. Immunol 136, 993–1006.e1 (2015). 26162572 10.1016/j.jaci.2015.05.036 PMC5042203

[81] Okada S, Ishikawa N, Shirao K, Kawaguchi H, Tsumura M, Ohno Y, Yasunaga S, Ohtsubo M, Takihara Y, Kobayashi M, The novel IFNGR1 mutation 774del4 produces a truncated form of interferon-γ receptor 1 and has a dominant-negative effect on interferon-γ signal transduction. J. Med. Genet 44, 485–491 (2007). 17513528 10.1136/jmg.2007.049635 PMC2597923

[82] Ma CS, Wong N, Rao G, Nguyen A, Avery DT, Payne K, Torpy J, O’Young P, Deenick E, Bustamante J, Puel A, Okada S, Kobayashi M, Martinez-Barricarte R, Elliott M, Sebnem Kilic S, El Baghdadi J, Minegishi Y, Bousfiha A, Robertson N, Hambleton S, Arkwright PD, French M, Blincoe AK, Hsu P, Campbell DE, Stormon MO, Wong M, Adelstein S, Fulcher DA, Cook MC, Stepensky P, Boztug K, Beier R, Ikinciogullari A, Ziegler JB, Gray P, Picard C, Boisson-Dupuis S, Phan TG, Grimbacher B, Warnatz K, Holland SM, Uzel G, Casanova JL, Tangye SG, Unique and shared signaling pathways cooperate to regulate the differentiation of human CD4+ T cells into distinct effector subsets. J Exp Med. 213, 1589–1608 (2016). 27401342 10.1084/jem.20151467 PMC4986526

[83] Hirata O, Okada S, Tsumura M, Kagawa R, Miki M, Kawaguchi H, Nakamura K, Boisson-Dupuis S, Casanova JL, Takihara Y, Kobayashi M, Heterozygosity for the Y701C STAT1 mutation in a multiplex kindred with multifocal osteomyelitis. Haematologica. 98, 1641–1649 (2013). 23585529 10.3324/haematol.2013.083741 PMC3789471

[84] Sakata S, Tsumura M, Matsubayashi T, Karakawa S, Kimura S, Tamaura M, Okano T, Naruto T, Mizoguchi Y, Kagawa R, Nishimura S, Imai K, Le Voyer T, Casanova JL, Bustamante J, Morio T, Ohara O, Kobayashi M, Okada S, Autosomal recessive complete STAT1 deficiency caused by compound heterozygous intronic mutations. Int. Immunol 32, 663–671 (2020). 32603428 10.1093/intimm/dxaa043

[85] Picard C, Von Bernuth H, Ghandil P, Chrabieh M, Levy O, Arkwright PD, McDonald D, Geha RS, Takada H, Krause JC, Creech CB, Ku CL, Ehl S, Maródi L, Al-Muhsen S, Al-Hajjar S, Al-Ghonaium A, Day-Good NK, Holland SM, Gallin JI, Chapel H, Speert DP, Rodriguez-Gallego C, Colino E, Garty BZ, Roifman C, Hara T, Yoshikawa H, Nonoyama S, Domachowske J, Issekutz AC, Tang M, Smart J, Zitnik SE, Hoarau C, Kumararatne DS, Thrasher AJ, Davies EG, Bethune C, Sirvent N, De Ricaud D, Camcioglu Y, Vasconcelos J, Guedes M, Vitor AB, Rodrigo C, Almazán F, Méndez M, Aróstegui JI, Alsina L, Fortuny C, Reichenbach J, Verbsky JW, Bossuyt X, Doffinger R, Abel L, Puel A, Casanova JL, Clinical features and outcome of patients with IRAK-4 and MyD88 deficiency. Medicine (Baltimore). 89, 403–425 (2010). 21057262 10.1097/MD.0b013e3181fd8ec3 PMC3103888

[86] Corces MR, Trevino AE, Hamilton EG, Greenside PG, Sinnott-Armstrong NA, Vesuna S, Satpathy AT, Rubin AJ, Montine KS, Wu B, Kathiria A, Cho SW, Mumbach MR, Carter AC, Kasowski M, Orloff LA, Risca VI, Kundaje A, Khavari PA, Montine TJ, Greenleaf WJ, Chang HY, An improved ATAC-seq protocol reduces background and enables interrogation of frozen tissues. Nat. Methods 14, 959–962 (2017). 28846090 10.1038/nmeth.4396 PMC5623106

[87] Benet ZL, Marthi M, Ke F, Wu R, Turner JS, Gabayre JB, Ivanitskiy MI, Sethi SS, Grigorova IL, CCL3 promotes germinal center B cells sampling by follicular regulatory T cells in murine lymph nodes. Front. Immunol 9 (2018), doi:10.3389/fimmu.2018.02044. PMC6146081 30271404

[88] Unutmaz D, Vilcek J, IRF1: A deus ex machina in TH1 differentiation. Nat. Immunol 9, 9–10 (2008). 18087248 10.1038/ni0108-9

[89] Kano S, Sato K, Morishita Y, Vollstedt S, Kim S, Bishop K, Honda K, Kubo M, Taniguchi T, The contribution of transcription factor IRF1 to the interferon-γ–interleukin 12 signaling axis and TH1 versus TH-17 differentiation of CD4+ T cells. Nat. Immunol 9, 34–41 (2008). 18059273 10.1038/ni1538

[90] Desnues B, Macedo AB, Ordoñez-Rueda D, Roussel-Queval A, Malissen B, Bruhns P, Malissen M, Alexopoulou L, The transcriptional repressor Gfi1 prevents lupus autoimmunity by restraining TLR7 signaling. Eur. J. Immunol 46, 2801–2811 (2016). 27600904 10.1002/eji.201646573

[91] Jackson SW, Jacobs HM, Arkatkar T, Dam EM, Scharping NE, Kolhatkar NS, Hou B, Buckner JH, Rawlings DJ, B cell IFN-γ receptor signaling promotes autoimmune germinal centers via cell-intrinsic induction of BCL-6. J. Exp. Med 213, 733–750 (2016). 27069113 10.1084/jem.20151724 PMC4854732

[92] Winkelstein JA, Marino MC, Lederman HM, Jones SM, Sullivan K, Burks AW, Conley ME, Cunningham-Rundles C, Ochs HD, X-linked agammaglobulinemia: Report on a United States registry of 201 patients. Medicine (Baltimore). 85, 193–202 (2006). 16862044 10.1097/01.md.0000229482.27398.ad

[93] Tangye SG, Al-Herz W, Bousfiha A, Chatila T, Cunningham-Rundles C, Etzioni A, Franco JL, Holland SM, Klein C, Morio T, Ochs HD, Oksenhendler E, Picard C, Puck J, Torgerson TR, Casanova JL, Sullivan KE, Human Inborn Errors of Immunity: 2019 Update on the Classification from the International Union of Immunological Societies Expert Committee. J. Clin. Immunol 40, 24–64 (2020). 31953710 10.1007/s10875-019-00737-x PMC7082301

[94] Singh M, Jackson KJL, Wang JJ, Schofield P, Field MA, Koppstein D, Peters TJ, Burnett DL, Rizzetto S, Nevoltris D, Masle-Farquhar E, Faulks ML, Russell A, Gokal D, Hanioka A, Horikawa K, Colella AD, Chataway TK, Blackburn J, Mercer TR, Langley DB, Goodall DM, Jefferis R, Gangadharan Komala M, Kelleher AD, Suan D, Rischmueller M, Christ D, Brink R, Luciani F, Gordon TP, Goodnow CC, Reed JH, Lymphoma Driver Mutations in the Pathogenic Evolution of an Iconic Human Autoantibody. Cell. 180, 878–894.e19 (2020). 32059783 10.1016/j.cell.2020.01.029

[95] Magoč T, Salzberg SL, FLASH: Fast length adjustment of short reads to improve genome assemblies. Bioinformatics. 27, 2957–2963 (2011). 21903629 10.1093/bioinformatics/btr507 PMC3198573

[96] Vander Heiden JA, Yaari G, Uduman M, Stern JNH, O’connor KC, Hafler DA, Vigneault F, Kleinstein SH, PRESTO: A toolkit for processing high-throughput sequencing raw reads of lymphocyte receptor repertoires. Bioinformatics. 30, 1930–1932 (2014). 24618469 10.1093/bioinformatics/btu138 PMC4071206

[97] Ye J, Ma N, Madden TL, Ostell JM, IgBLAST: an immunoglobulin variable domain sequence analysis tool. Nucleic Acids Res. 41 (2013), doi:10.1093/nar/gkt382. PMC3692102 23671333

[98] Fu L, Niu B, Zhu Z, Wu S, Li W, CD-HIT: Accelerated for clustering the next-generation sequencing data. Bioinformatics. 28, 3150–3152 (2012). 23060610 10.1093/bioinformatics/bts565 PMC3516142

[99] Avery DT, Deenick EK, Ma CS, Suryani S, Simpson N, Chew GY, Chan TD, Palendira U, Bustamante J, Boisson-Dupuis S, Choo S, Bleasel KE, Peake J, King C, French MA, Engelhard D, Al-Hajjar S, Al-Muhsen S, Magdorf K, Roesler J, Arkwright PD, Hissaria P, Riminton DS, Wong M, Brink R, Fulcher DA, Casanova JL, Cook MC, Tangye SG, B cell-intrinsic signaling through IL-21 receptor and STAT3 is required for establishing long-lived antibody responses in humans. J. Exp. Med 207, 155–171 (2010). 20048285 10.1084/jem.20091706 PMC2812540

[100] Rosain J, Deswarte C, Hancioglu G, Oleaga-Quintas C, Kutlug S, Kartal I, Kuzu I, Toullec L, Tusseau M, Casanova JL, Yildiran A, Bustamante J, LINE-1-Mediated AluYa5 Insertion Underlying Complete Autosomal Recessive IFN-γR1 Deficiency. J. Clin. Immunol 39 (2019), pp. 739–742. 31377971 10.1007/s10875-019-00667-8 PMC8165577

[101] Taramasso L, Boisson-Dupuis S, Garrè ML, Bondi E, Cama A, Nozza P, Morana G, Casanova JL, Marazzi MG, Pineal Germinoma in a Child with Interferon-γ Receptor 1 Deficiency. Case Report and Literature Review. J. Clin. Immunol 34, 922–927 (2014). 25216720 10.1007/s10875-014-0098-0

[102] Marazzi MG, Chapgier A, Defilippi AC, Pistoia V, Mangini S, Savioli C, Dell’Acqua A, Feinberg J, Tortoli E, Casanova JL, Disseminated Mycobacterium scrofulaceum infection in a child with interferon-γ receptor 1 deficiency. Int. J. Infect. Dis 14 (2010), doi:10.1016/j.ijid.2009.03.025. 19880337

[103] Korsunsky I, Millard N, Fan J, Slowikowski K, Zhang F, Wei K, Baglaenko Y, Brenner M, ru Loh P, Raychaudhuri S, Fast, sensitive and accurate integration of single-cell data with Harmony. Nat. Methods 16, 1289–1296 (2019). 31740819 10.1038/s41592-019-0619-0 PMC6884693

[104] Buenrostro JD, Wu B, Chang HY, Greenleaf WJ, Curr Protoc Mol Biol, in press, doi:10.1002/0471142727.mb2129s109. PMC4374986 25559105

[105] Buenrostro JD, Giresi PG, Zaba LC, Chang HY, Greenleaf WJ, Transposition of native chromatin for fast and sensitive epigenomic profiling of open chromatin, DNA-binding proteins and nucleosome position. Nat Methods. 10, 1213–1218 (2013). 24097267 10.1038/nmeth.2688 PMC3959825

[106] Liao Y, Smyth GK, Shi W, featureCounts: an efficient general purpose program for assigning sequence reads to genomic features. Bioinformatics. 30, 923–930 (2014). 24227677 10.1093/bioinformatics/btt656

[107] Lawrence M, Gentleman R, Carey V, rtracklayer: An R package for interfacing with genome browsers. Bioinformatics. 25, 1841–1842 (2009). 19468054 10.1093/bioinformatics/btp328 PMC2705236

[108] Feng J, Liu T, Qin B, Zhang Y, Liu XS, Identifying ChIP-seq enrichment using MACS. Nat. Protoc 7, 1728–1740 (2012). 22936215 10.1038/nprot.2012.101 PMC3868217

[109] Zhang Y, Liu T, Meyer CA, Eeckhoute J, Johnson DS, Bernstein BE, Nussbaum C, Myers RM, Brown M, Li W, Liu XS, Model-based Analysis of ChIP-Seq (MACS). Genome Biol. 9, R137 (2008). 18798982 10.1186/gb-2008-9-9-r137 PMC2592715

[110] Carroll TS, Liang Z, Salama R, Stark R, de Santiago I, Impact of artifact removal on ChIP quality metrics in ChIP-seq and ChIP-exo data. Front. Genet 5, 75 (2014). 24782889 10.3389/fgene.2014.00075 PMC3989762

[111] de Santiago I, Carroll T, (2018; http://link.springer.com/10.1007/978-1-4939-7380-4_17), pp. 195–226. 10.1007/978-1-4939-7380-4_17 29027176

[112] Love MI, Huber W, Anders S, Moderated estimation of fold change and dispersion for RNA-seq data with DESeq2. Genome Biol. 15, 550 (2014). 25516281 10.1186/s13059-014-0550-8 PMC4302049

[113] Ma W, Noble WS, Bailey TL, Motif-based analysis of large nucleotide data sets using MEME-ChIP. Nat. Protoc 9, 1428–1450 (2014). 24853928 10.1038/nprot.2014.083 PMC4175909

