# Supplementary material for: Human T-bet governs the generation of a distinct subset of CD11chighCD21low B cells
Source: Sci Immunol. Author manuscript; Available in PMC 2023 Jan 22. (PMC9413977; doi:10.1126/sciimmunol.abq3277)
Supplement: main supplementary — Figure S1. Polyclonal and antigen-specific antibody responses in inherited T-bet deficiency. Figure S2. Characterization of the CD21loCD11c+ B-cell subset. Figure S3. Unsupervised FlowSOM analysis of B cells from a patient with inherited human T-bet deficiency. Figure S4. Enrichment of IgG- or IgA-expressing B cells in CD21loCD11c+ B cells. Figure S5. Proteotranscriptomic investigation of CD21lo B cells from a patient with inherited human T-bet deficiency. Figure S6. Chromatin accessibilities of B cells are altered in inherited T-bet deficiency. Table S1. Vaccine-specific antibody responses in T-bet deficiency. Plasma samples prepared when the patient was 6-month-, 2-year-, and 3-year-old were measured for the levels of antigen-specific antibodies. Units are IU/mL if not otherwise specified. ND not detectable. [file NIHMS1828447-supplement-main_supplementary.docx]

**Human T-bet governs the generation of a distinct subset**

**of CD11c^high^CD21^low^ B cells**

**Supplementary Material**

Supplementary Methods

Figure S1. Polyclonal and antigen-specific antibody responses in inherited T-bet deficiency.

Figure S2. Characterization of the CD21^lo^CD11c^+^ B-cell subset.

Figure S3. Unsupervised FlowSOM analysis of B cells from a patient with inherited human T-bet deficiency.

Figure S4. Enrichment of IgG- or IgA-expressing B cells in CD21^lo^CD11c^+^ B cells.

Figure S5. Proteotranscriptomic investigation of CD21^lo^ B cells from a patient with inherited human T-bet deficiency.

Figure S6. Chromatin accessibilities of B cells are altered in inherited T-bet deficiency.

Table S1. Vaccine-specific antibody responses in T-bet deficiency.

Data file S1. CITE-seq of CD21^lo^ B cells.

Data file S2. ATAC-seq of CD21^lo^ B cells.

Data file S3. List of Cite-seq and ATAC-seq samples.

Data file S4. Raw data file (Excel spreadsheet)

Reproducibility checklist.

**Supplementary Methods**

**Immunophenotyping for B cells with conventional flow cytometry**

The following mAbs were used: BV711-anti CD19, FITC-anti CD20, BUV395-anti CD20, PE-anti CD27, PE-Cy7-anti CD27, BV786-anti CD27, APC-anti CD10, BV421-anti CD21, BV605-anti CD80, BUV737-anti CD86, PE-CF595-anti CD95, APC-anti CXCR3 (CD183), PE-CF594-anti CXCR3 (CD183), BV421-anti CXCR5 (CD185), PE-anti TACI (CD267), FITC-anti BAFF-RR (CD268), APC-anti human IgG, BV605-anti human IgG, biotinylated-anti-IgG1, biotinylated-anti-IgG2, biotinylated-anti-IgG4, and PerCP-Cy5.5 Streptavidin from Becton Dickinson. Zombie Aqua Fixable Viability dye, Pacific Blue-anti CD20, APC-Cy7-anti CD11c, BV421-anti CXCR3 (CD183), PE-anti FcRL5 (CD307e), and PerCP-Cy5.5-anti T-bet antibodies were obtained from BioLegend. FITC-anti CCR7 (CD197) antibody was obtained from R&D Systems. Biotinylated-anti-IgG3 was from Sigma Aldrich. PBMCs were labeled with mAbs specific for CD20, CD19, CD21, CD10, CD27, IgG and IgA. Proportions of transitional (CD20^+^CD10^+^CD27^-^), naïve (CD20^+^CD10^-^CD27^-^); memory (CD20^+^CD10^-^CD27^+^), and CD21^lo^CD19^hi^ cells and of memory B cells expressing IgG, IgG subclasses (IgG1, 2, 3 4) or IgA were determined. We characterized CD21^lo^CD19^hi^ B cells further, by incubating PBMCs with mAbs directed against CD19, CD20, CD21, CCR7, CD11c, CD80, CD86, CXCR3, FcRL5, CD95, BAFF-R, and TACI, then fixing and permeabilizing them with the True-Nuclear Transcription Factor Buffer Set (BioLegend), before labeling with anti-T-bet mAb. The expression of CD19, CD20, CD21, CCR7, CD11c, CD80, CD86, CXCR3, FcRL5, CD95, BAFF-R, TACI and T-bet by CD21^lo^CD19^hi^ and CD19^+^CD21^+^ B cells was determined by flow cytometry (LSRFortessa; Becton Dickinson) and analyzed with FlowJo software (Tree Star).

**Unsupervised analysis of data from spectral flow cytometry**

Data from two batches of experiments were concatenated through the iMUBAC pipeline, as previously described (*69*). In brief, the marker expression values of live single B lymphocytes (CD3^-^CD56^-^CD19^+^CD20^+^) (manually gated on FlowJo) were first imported into R. Data from healthy adult controls (randomly downsampled to 50,000 cells per batch) were then batch-corrected with Harmony (*103*). The batch-corrected expression values for all the available markers except T-bet, IgG, IgM, and IgD were then used for FlowSOM-guided unsupervised clustering analysis. T-bet was excluded during clustering analysis, to ensure that T-bet-expressing and T-bet-deficient cells were treated in an unbiased manner. Because IgG-PE/Cy5 signal was almost undetectable due to an uncommon technical mistake, all surface Ig markers including IgM, IgD, and IgG were excluded from the analysis. In total, 30 clusters were generated using unsupervised algorithm. Cluster labels were then back-propagated to all cells from both controls and patients, through batch-specific machine learning. For visualization, the clusters representing dead cells and contaminating T and NK lymphocytes were excluded, and all cells from both batches were then batch-corrected with Harmony (*103*). The batch-corrected cells (randomly downsampled to 20,000 cells per individual) from age-matched controls, P's healthy brother and mother, P, and the STAT1-deficient patient were used for dimension reduction through uniform manifold approximation and projection (UMAP). All analyses were performed in R v4.0 (<https://www.r-project.org/>).

**Immunophenotyping of surface B cell receptors with spectral flow cytometry**

PBMCs were obtained from seventeen healthy adult donors, one age-matched control (3 years of age at the time of sampling), P (6 years of age at the time of sampling), P's healthy brother (10 years of age at the time of sampling), who is wild-type for the *TBX21* locus, and P’s healthy mother, who is heterozygous for the mutation (*55*, *100*–*102*). P’s sample was split into three separate staining samples, P’s healthy brother sample and P’s healthy mother sample were split into two independent staining samples each as technical replicates. We stained 1.5 x 10^6^ to 4 x 10^6^ PBMCs from each individual with Zombie-NIR live-dead exclusion dye (BioLegend). Cells were then labeled with anti-IgG-PE/Cy5 (BD Biosciences), anti-IgA-Viogreen (Miltenyi Biotec), anti-IgM-BV570 (BioLegend), and anti-IgD-BV785 (BioLegend) for 30 min. Cells were then stained with FcBlock (Miltenyi Biotec) and antibodies (Abs) against surface antigens, including anti-CD10-BUV737 (BD Biosciences), anti-CD23-BUV805 (BD Biosciences), anti-CD80-FITC (BioLegend), anti-FcRL4-PERCP/Cy5.5 (BioLegend), anti-CD138-PE/Dazzle594 (BioLegend), anti-FcRL5-APC (BioLegend), anti-CD269-APC/Fire750 (BioLegend), anti-CXCR3-BUV496 (BD Biosciences), anti-CD20-Alexa532 (Thermo Fisher Scientific), anti-CD11b-BUV395 (BD Biosciences), anti-CD38-BUV661 (BD Biosciences), anti-CD24-BUV563 (BD Biosciences), anti-CD56-V450 (BD Biosciences), anti-CD3-V450 (BD Biosciences), anti-CD5-BV480 (BD Biosciences), anti-CD95-BV605 (BioLegend), anti-HLA-DR-PE-Fire810 (BioLegend), anti-CD40-BV650 (BioLegend), anti-CD21-PE (BD Biosciences), anti-CD86-Alexa647 (BioLegend), anti-CD11c-Alexa700 (BioLegend), anti-CD27-BV711 (BioLegend), anti-CD71-BV750 (BD Biosciences), anti-CXCR4-BV421 (BioLegend), and anti-CD19-Spark/NIR685 (BioLegend) antibodies. The cells were then fixed and permeabilized with the FOXP3/Transcription factor staining buffer set (eBioscience). Cells were subjected to intracellular staining with anti-T-bet-PE/Cy7 antibody overnight (BioLegend). Data were acquired by spectral flow cytometry (Cytek). Manual gating and statistical analyses were performed with Cytobank.

**Real-time quantitative *ENC1* PCR (qPCR).**

Total RNA was isolated from transitional, naïve, memory and CD21^lo^CD19^hi^ B cells purified by FACS-sorting from the peripheral blood of healthy donors. A mixture of random octamers and oligo dT-16 was used, with MultiScribe reverse transcriptase (High-Capacity RNA-to-cDNA kit, Thermo Fisher Scientific), to generate cDNA. Quantitative real-time PCR was performed with the TaqMan Universal PCR Master Mix (Thermo Fisher Scientific), *ENC1*-specific FAM-MGB primer (Hs00171580_m1, Thermo Fisher Scientific) and either endogenous human *GUSB* (43626320EE) or *HPRT1* (4326321E, Thermo Fisher Scientific) probes. Data were analyzed by the ΔΔCt method, with normalization against *GUSB* or *HPRT1*.

**Analysis of CITE-seq data**

The data for this study have been deposited in database NCBI SRA. The sequence of CITE-seq can be found as PRJNA835417 (**Data file S3**). Cellranger counts [10X] were used for mapping and gene/HTO counting according to the protocol described in the 10X Genomics manual, with default parameters. The human v38 reference sequence was downloaded from the 10X Genomics website and uncompressed. The counting matrix generated by cellranger was imported into Seurat for the downstream analysis. Doublet cells were detected and removed with the Scrublet package, using the default parameters. Cells with more than 10% mitochondrial reads, or fewer than 200 or more than 6000 genes expressed were filtered out. HTO and BCR matrix of the cellranger output are split according to the TotalSeq barcode and the BCR barcode. HTOs and BCR were assigned to the sample IDs by the Seurat function HTODemux, respectively, using the default parameters. After the QC steps, Expression data for 3557 housekeeping genes were extracted from the original matrix and a new Seurat object for clustering was built. Both Seurat objects (one for the matrix of all genes, and the other for housekeeping genes only) followed the default Seurat pipeline for normalization and scale. The cells were clustered into five groups with a resolution of 0.25. After the removal of apoptotic cells, identified on the basis of the high proportion of mitochondrial reads, and the extraction of reads from individual hashtags, we performed unbiased clustering of the 7039 cells available for analysis. They formed three clusters based on the expression of the 3557 housekeeping genes. Cells from an IFN-γR1-deficient patient (#15553) formed a distant outlier cluster. Cells from two age-matched controls, another IFN-γR1-deficient patient (#1114), and P formed housekeeping cluster B. Cells from two other age-matched controls, the other IFN-γR1-deficient patient (#13391), and P’s brother formed housekeeping cluster C. As the PBMCs from these nine individuals were processed and frozen separately, these three clusters based on housekeeping genes are probably due to batch artifacts related to sample quality. We minimized batch effects, by focusing on comparisons of the cells from P with those from the three other individuals from the same cluster (house-keeping cluster B), which included two age-matched controls, an IFN-γR1-deficient patient, and the T-bet-deficient patient.

**Single-cell VDJ sequencing analysis**

Cellranger VDJ was applied to the VDJ sequencing data with the reference version GRCh38-alts-ensembl-5.0.0 downloaded from 10X Genomics. All contig table from the cellranger vdj output were selected for the downstream analysis. Briefly, we characterized a single pair of junctional sequences (CDR3) for both the Ig heavy and light chains, without permitting any mismatches, as a clonotype. The IgM, IgD, IgE, IgA1, IgA2, IgG1, IgG2, IgG3, and IgG4 annotations were extracted from the contig table. As 10x VDJ cell ranger annotates only one of the IgM or IgD chains from each individual cell, cells annotated as IGHM or IGHD were categorized as unswitched B cells. We determined mutation frequency, reflecting the level of somatic hypermutation, at single-cell level, by selecting a 280-nt region located from -21 bp to -300 bp relative to the first nucleotide of the CDR3 sequence. The selection of this region minimized 1) the frequency of artifactual assembly errors by 10x VDJ cellranger at the 5’ end of the assembled sequence; and 2) undercoverage of the CDR3 region, potentially resulting in the omission of mutations proximal to the 5’ end of CDR3. The number of mutations was counted, using the inferred germline sequence as the template. Mutation frequency was calculated by dividing the number of mutations by 280. A mutation frequency of 0.01% or 1% was used as a cutoff for data presentation.

**Naïve B-cell differentiation for Omni-ATAC-seq**

Frozen PBMCs from five healthy adults, P (M/M), and P’s healthy brother (WT/WT) were used for this experiment. PBMCs were thawed, washed, and resuspended in Zombie NIR diluted in PBS (BioLegend). After 15 minutes of incubation at room temperature, the cells were labeled by incubation for 20 minutes with FcBlock (Miltenyi Biotec), anti-IgG-PE/Cy5 (BD Biosciences), anti-CD27-BV711 (BioLegend), anti-CD10-APC (BioLegend), anti-CD56-V450 (BD Biosciences), anti-CD3-V450 (BD Biosciences), anti-CD20-FITC (BioLegend), anti-CD21-PE (BD Biosciences), and anti-CD11c-Alexa700 (BioLegend) antibodies. The cells were then washed and subjected to FACS sorting. Live CD20^+^CD3^-^CD56^-^CD27^-^IgG^-^CD10^-^ naïve B cells were isolated from each PBMC sample. Naïve B cells were resuspended in 10% FBS in RPMI, and the volume was topped up to achieve a density for each sample of 35,000 cells to 55,000 cells per 200 µL. We plated 200 µL of the cell suspension in a single well of a 96-well U-bottomed plate. Samples were plated in triplicate. One set of wells was left untreated. Another set of wells was stimulated with 0.83 µg/mL anti-Ig Fab (Jackson ImmunoResearch Laboratories), 0.33 µg/mL CpG2006 (Sigma-Aldrich), and 50 ng/mL recombinant IFN-γ (Becton Dickinson). The last set of wells was stimulated with 0.83 µg/mL anti-Ig Fab (Jackson ImmunoResearch Laboratories), 0.33 µg/mL CpG2006 (Sigma-Aldrich), and 100 ng/mL recombinant IL-27 (PeproTech). After stimulation for 1 day, cells were harvested for Omni-ATAC-seq.

Omni-ATAC-seq library preparation was performed as previously described (*55*, *86*). Briefly, about 25,000 cells from each well were harvested, washed with 50 µL cold PBS, and centrifuged to obtain a pellet. Pellets were lysed with 50 µl cold lysis buffer consisting of 48.5 µL resuspension buffer (10 mM Tris-HCl pH 7.5, 10 mM NaCl, 3 mM MgCl_2_ in water), 0.5 µL 10% NP-40, 0.5 µL 10% Tween-20, and 0.5 µL 1% digitonin. Lysates were incubated on ice and then washed with 0.1% Tween-20 resuspension buffer and centrifuged. The nuclei in the pellet were subjected to Tn5 transposition with 20 µL of a mixture of 10 µL 2 x TD buffer, 6.6 µL PBS, 0.2 µL 10% Tween-20, 0.2 µL 1% digitonin, 1 µL Tn5 transposase, 2 µL nuclease-free water at 37 °C in a thermomixer operating at 1,000 rpm for 30 minutes. DNA fragments were extracted with the MinElute PCR purification kit and eluted in 20 µL EB buffer (Qiagen). We used 5 µL of eluted DNA for the amplification of DNA fragments with the NEBNext PCR master mix (New England Biolabs), over six cycles, with the Ad1_forward and 15 indexed Ad2_reverse primers, as previously described (*104*, *105*). We used an additional 5 µL of eluted DNA from the patient’s B cells (M/M) as a technical duplicate for amplification. The partially amplified library was subjected to quantitative PCR analysis with the Ad1 and Ad2 primers, SYBR Green reagents and the NEBNext PCR master mix for 25 cycles. Additional PCR cycles were performed, based on 1/3 the maximal fluorescence for each sample, as previously described (*104*, *105*). A left-sided isolation of PCR products was performed with AMPure beads (Beckman Coulter). DNA was quantified with a Qubit fluorimeter. Equal amounts of each sample were pooled and subjected to paired-end sequencing on a NovaSeq high-output sequencer to generate 50-bp pair-end reads.

**Analysis of Omni-ATAC-seq**

The data for this study have been deposited in database NCBI SRA. The sequence of ATAC-seq can be found as PRJNA835417 (**Data file S3**). The ATAC-seq reads were aligned with the hg38 genome sequence from the BSgenome.Hsapiens.UCSC.hg38 Bioconductor package (version 1.4.1) with Rsubread's align method in paired-end mode, with fragments between 1 and 5,000 base pairs long considered correctly paired (*106*). Normalized, fragment-extended bigWigs signals were created with the rtracklayer package (*107*). Peak calls were made with MACS2 software in BAMPE mode and predicted fragment lengths were calculated with the ChIPQC package (*108*–*111*). Heatmaps with normalized z-scores were generated with the pheatmap software package (https://CRAN.R-project.org/package=pheatmap). For differential accessibility analysis we imported raw ATAC-seq read counts into DESeq2 (version 1.20.0) and differential ATAC-seq signals were identified with the DESeq2 package (adjusted *p*-value < 0.05) (*112*). Peak summits that were shared by cells stimulated with αIg+CpG+IFN-γ and αIg+CpG+IL-27 were used in MEME-ChIP software for the identification of known and motif motifs (*113*). For the identification of regions in which chromatin accessibility differed between P and controls within each treatment group, samples from P were compared to the combined set of healthy controls and P’s healthy brother. For the identification of stimulation-dependent regions, the samples from each individual (P, P’s brother, or control) were treated as paired samples across all treatments (αIg+CpG+IL-27 vs. non-stimulated, or αIg+CpG+IFN-γ vs. non-stimulated).

**
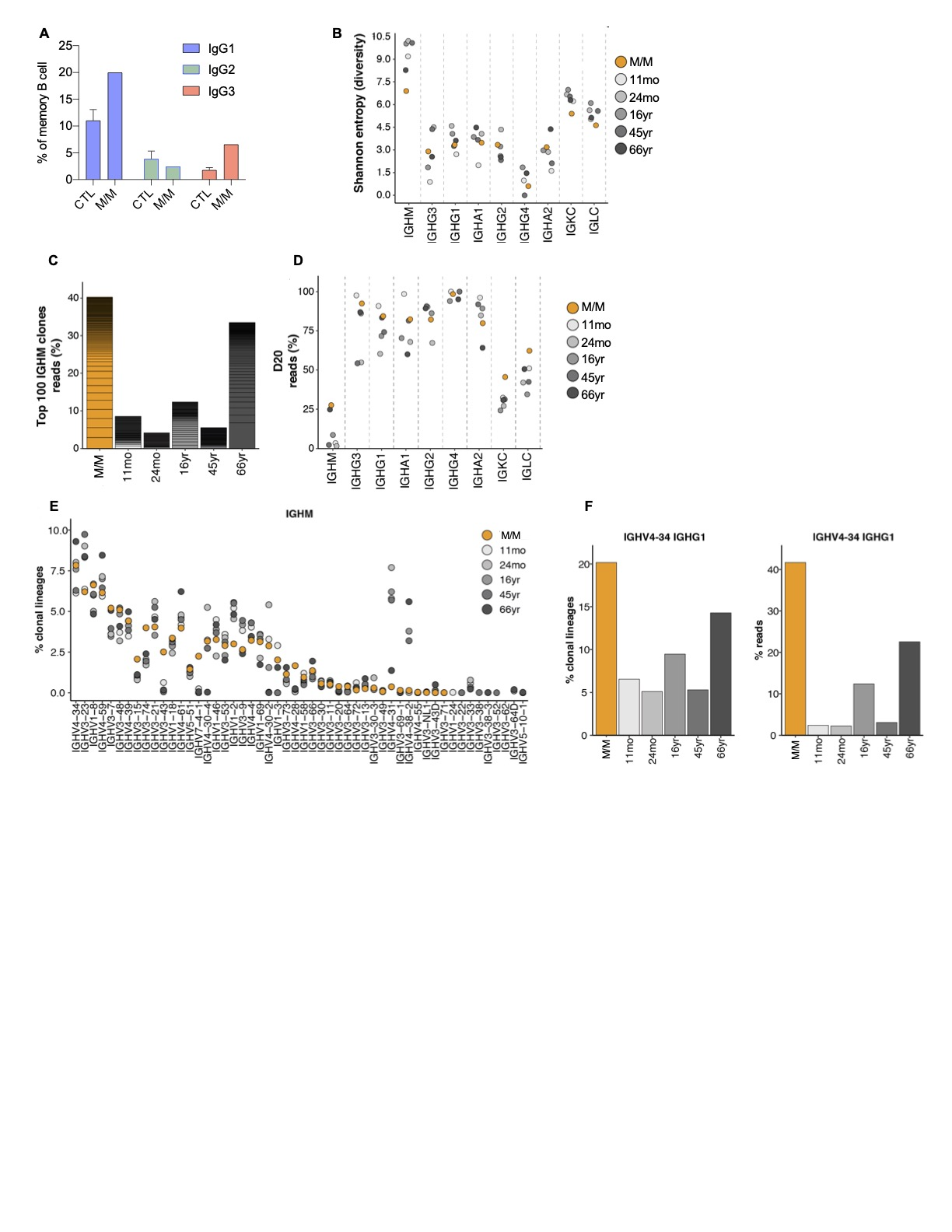
**

**Figure S1. Polyclonal and antigen-specific antibody responses in inherited T-bet deficiency. (A)** Frequencies of IgG1-, IgG2-, or IgG3-expressing memory B cells in healthy donors or patient with inherited T-bet deficiency (M/M). **(B)** Shannon entropy for each IGH isotype, IGK and IGL. **(C)** Contribution of the top 100 clones to the IgM repertoire for each donor. Each stacked section indicates a single clone. **(D)** D20 metric (the % of reads comprising the 20 largest clones) for each donor, for each isotype and the light chains. **(E)** IGHV gene usage for P (M/M) and controls for the IgM repertoire, expressed as the percentage of clones using each IGHV. Each point corresponds to a subject. The results are presented in descending order of mean usage across all donors. **(F)** IGHV4-34 usage for IgG1 in each donor; percentage clones and percentage reads.

**
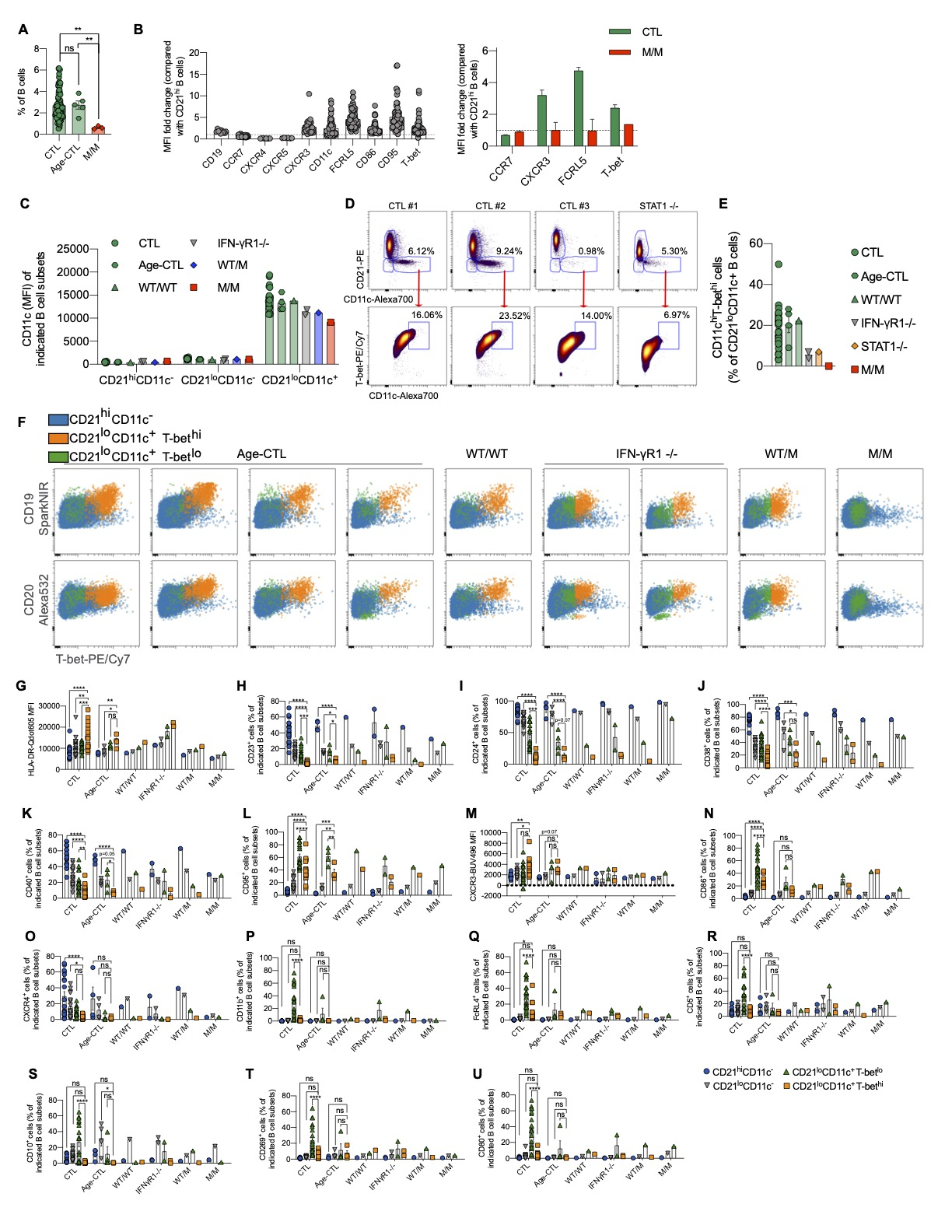
**

**Figure S2. Characterization of the CD21^lo^CD11c^+^ B-cell subset.** **(A)** PBMCs from adult healthy donors (CTL), age-matched healthy donors (Age-CTL), and P (M/M) were subjected to conventional flow cytometry. Frequency of CD21^lo^CD19^hi^ B cells were shown. (**B**) Relative fold change in the mean fluorescent intensities (MFIs) of indicated markers on CD21^lo^CD19^hi^ B cells were shown. Fold change was relative to the MFIs of those markers on CD21^hi^ B cells. (**C**) Mean fluorescence intensity of CD21^hi^CD11c^-^, CD21^lo^CD11c^-^, CD21^lo^CD11c^+^T-bet^lo^ B cells of adult controls (CTL), age-matched controls (Age-CTL), P with inherited T-bet deficiency (M/M), P’s wild-type brother (WT/WT), P’s heterozygous mom (WT/M), and two IFN-γR1-deficient patients (IFN-γR1 ^-/-^). (**D**) Spectral flow cytometry was performed to compare expression of indicated markers on control or STAT ^-/-^ PBMCs. Dot plots showing CD21^lo^CD11c^+^ and their T-bet and CD11c expression were shown. (**E**) Frequencies of T-bet^hi^CD11c^hi^ B cells, which expressed highest levels of T-bet and CD11c, among CD21^lo^CD11c^+^ B cells from indicated individuals. (**F**) CD21^hi^CD11c^-^ and CD21^lo^CD11c^+^ B cells, which were further divided into CD21^lo^CD11c^+^T-bet^lo^ and CD21^lo^CD11c^+^T-bet^hi^ B cells, were overlaid for their surface expression of CD19, CD20 and their intracellular levels of T-bet. **(G)** The expression levels of HLA-DR on CD21^hi^CD11c^-^, CD21^lo^CD11c^-^, CD21^lo^CD11c^+^T-bet^lo^ and CD21^lo^CD11c^+^T-bet^hi^ B cells, as represented by their MFI. **(H - L)** Frequencies of CD21^hi^CD11c^-^, CD21^lo^CD11c^-^, CD21^lo^CD11c^+^T-bet^lo^ and CD21^lo^CD11c^+^T-bet^hi^ B cells expressing high levels of CD23 (H), CD24 (I), CD38 (J), CD40 (K) and CD95 (L). **(M)** The expression levels of CXCR3 on CD21^hi^CD11c^-^, CD21^lo^CD11c^-^, CD21^lo^CD11c^+^T-bet^lo^ and CD21^lo^CD11c^+^T-bet^hi^ B cells, as indicated by their MFI values. **(N - U)** Frequencies of CD21^hi^CD11c^-^, CD21^lo^CD11c^-^, CD21^lo^CD11c^+^T-bet^lo^ and CD21^lo^CD11c^+^T-bet^hi^ B cells expressing high levels of CD86 (N), CXCR4 (O), CD11b (P), FcRL4 (Q), CD5 (R), CD10 (S), CD269 (T), and CD80 (U).

In Fig. 3A, B, C, and E, bars represent the mean and standard error of the mean. In Fig. 3G - U, bars represent the mean and standard deviation. Dots represent individual samples for CTL or Age-CTL and technical replicates for M/M. One-way ANOVA was performed to compare CTL, Age-CTL, and M/M (P) in (A). Mann-Whitney tests were performed to compare CD21^lo^CD11c^-^, CD21^lo^CD11c^+^T-bet^lo^, and CD21^hi^CD11c^-^ B cells with CD21^lo^CD11c^+^T-bet^hi^ B cells (G - U). In (A, G - T), **p*<0.05, ***p*<0.01, ****p*<0.001, *****p*<0.0001, and ns = not significant (or not marked).

**
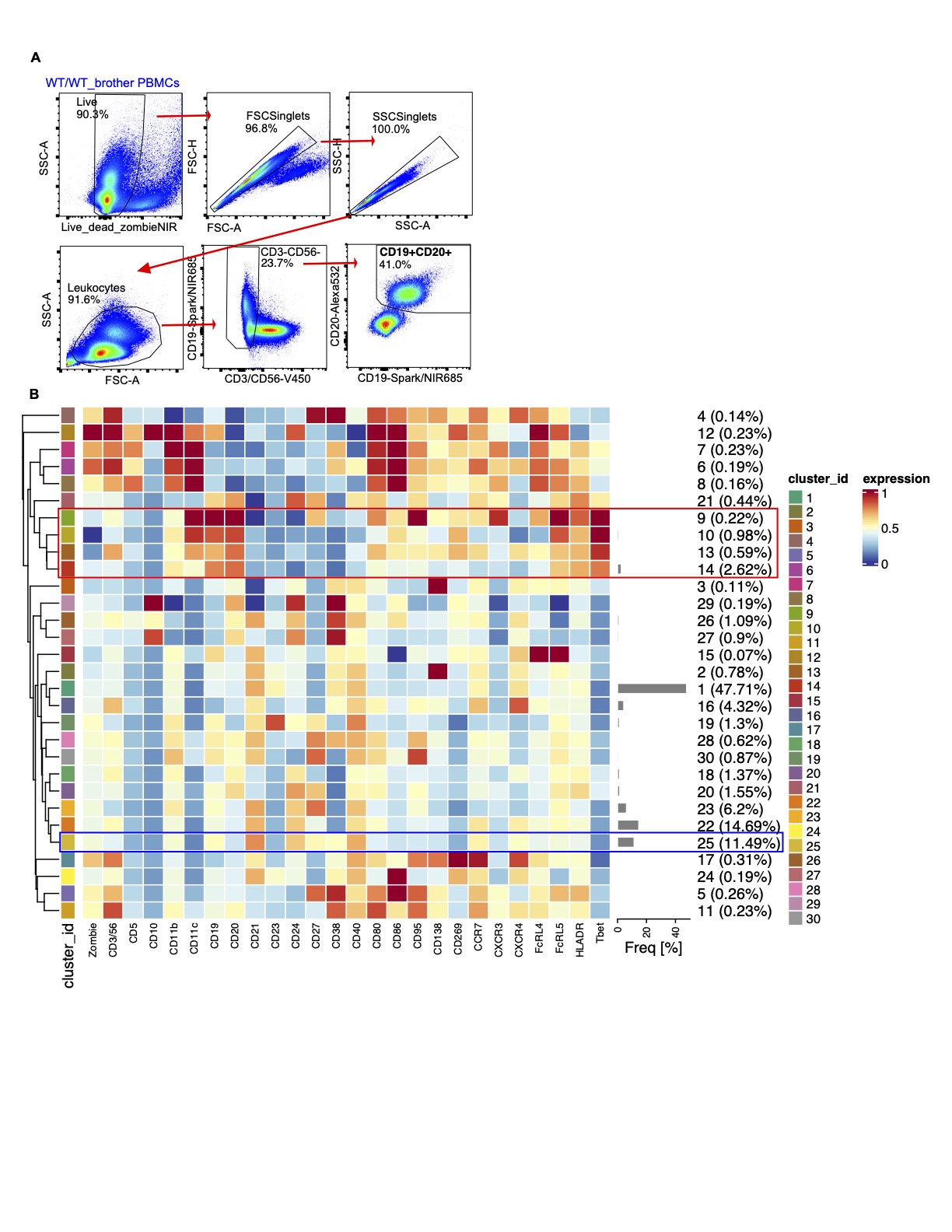
**

**Figure S3. Unsupervised FlowSOM analysis of B cells from a patient with inherited human T-bet deficiency. (A)** PBMCs from 30 healthy adults (CTL), four age-matched controls (Age-CTL), two IFN-γR1-deficient patients (IFN-γR1 ^-/-^), P's healthy brother (WT/WT), P’s heterozygous mother (WT/M), and P (M/M) were analyzed with a 29-color flow cytometry panel focusing on B cells in two separate experiments. The gating strategy used to identify B cells is shown. Batch-dependent differences in CD19^+^CD20^+^ B cells were corrected with the iMUBAC algorithm. Corrected datasets were compiled for FlowSOM analysis to form 30 self-organizing clusters based on B-cell markers except for T-bet, IgM, IgD, and IgG (clusters 1 to 30). **(B)** Summary heatmap of scaled median expression levels for all markers, for all cell subsets derived from CD3^-^CD56^-^CD19^+^CD20^+^ B cells.

**
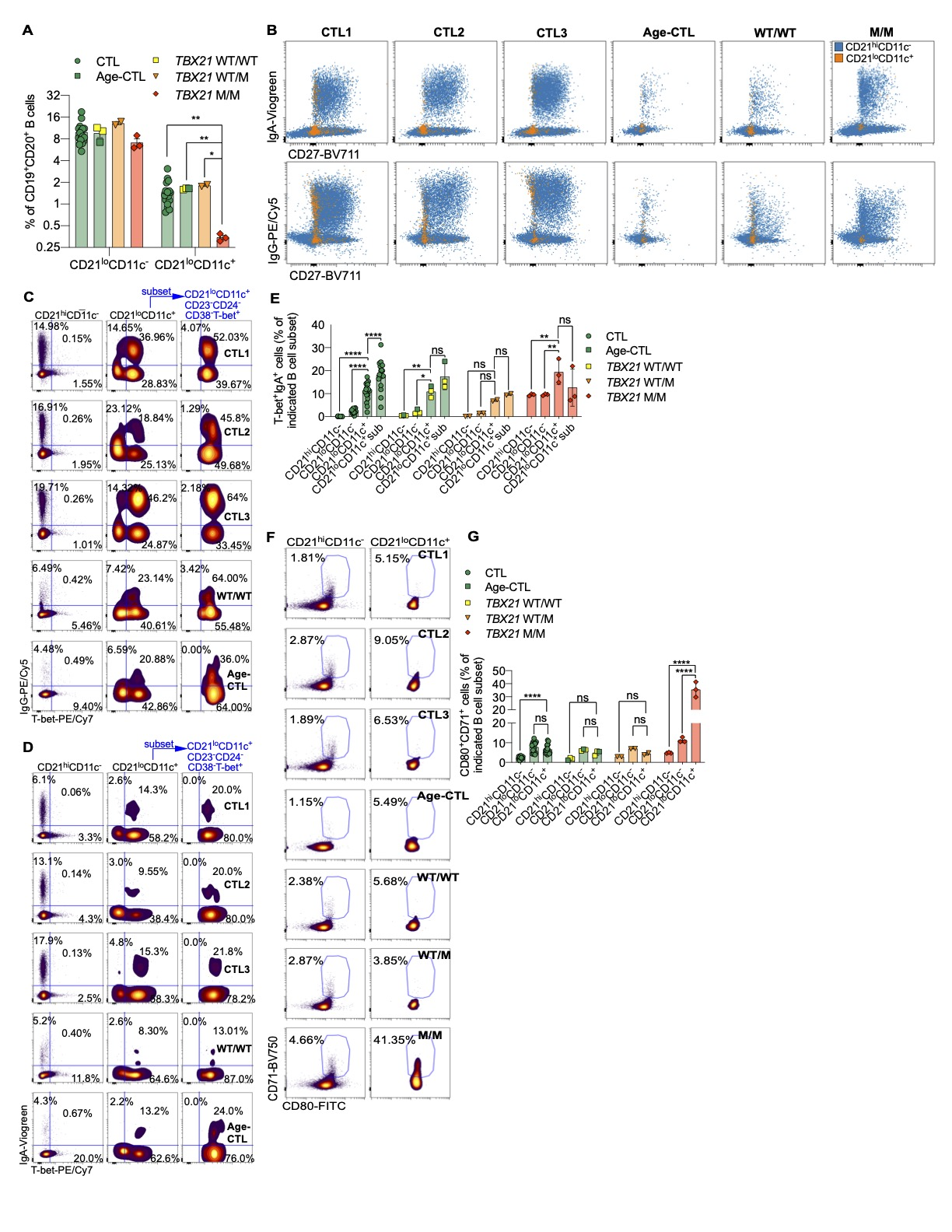
**

**Figure S4. Enrichment of IgG- or IgA-expressing B cells in CD21^lo^CD11c^+^ B cells. (A)** PBMCs from P (M/M), adult controls (CTL), age-matched controls (Age-CTL) including P's healthy brother (WT/WT), and heterozygous mother (WT/M) were analyzed with a flow cytometry panel including IgM, IgG, IgA, IgD, and CD71 staining focusing on B cells. Percentages of CD21^lo^CD11c^-^ and CD21^lo^CD11c^+^ B cells are shown. (**B**) CD21^hi^CD11c^-^ and CD21^lo^CD11c^+^ B cells, as in (A), were overlaid for their surface expression of IgA, IgG and CD27. **(C)** Surface expression of IgG and intracellular expression of T-bet, as in (A) were plotted. **(D)** Surface expression of IgA and intracellular expression of T-bet, as in (A) were plotted. **(E)** Percentages of T-bet^+^IgA^+^ among indicated subsets of B cells, as in (A), were shown. CD21^lo^CD11c^+^ sub represents CD21^lo^CD11c^+^CD23^-^CD24^-^CD38^-^T-bet^hi^ B cells. **(F)** Surface expression of CD71 and CD80, as in (A) were plotted. **(G)** Percentages of CD80^+^CD71^+^ among indicated subsets of B cells, as in (F), were shown. CD21^lo^CD11c^+^ sub represents CD21^lo^CD11c^+^CD23^-^CD24^-^CD38^-^T-bet^hi^ B cells.

In Fig. S4A, E, and G, bars represent the mean and the standard deviation. Dots represent individual samples for CTL or Age-CTL and technical replicates for M/M. One-way ANOVA with multiple comparison was used to compare CTL, Age-CTL, or WT/M against M/M in (A). One-way ANOVA with multiple comparison tests were performed to compare CD21^hi^CD11c^-^, CD21^lo^CD11c^-^, CD21^lo^CD11c^+^, and CD21^lo^CD11c^+^CD23^-^CD24^-^CD38^-^T-bet^hi^ (CD21^lo^CD11c^+^ sub) B cells against each other in (E and G). In (A, E, and G), **p*<0.05, ***p*<0.01, ****p*<0.001, *****p*<0.0001, and ns = not significant (or not marked).

**
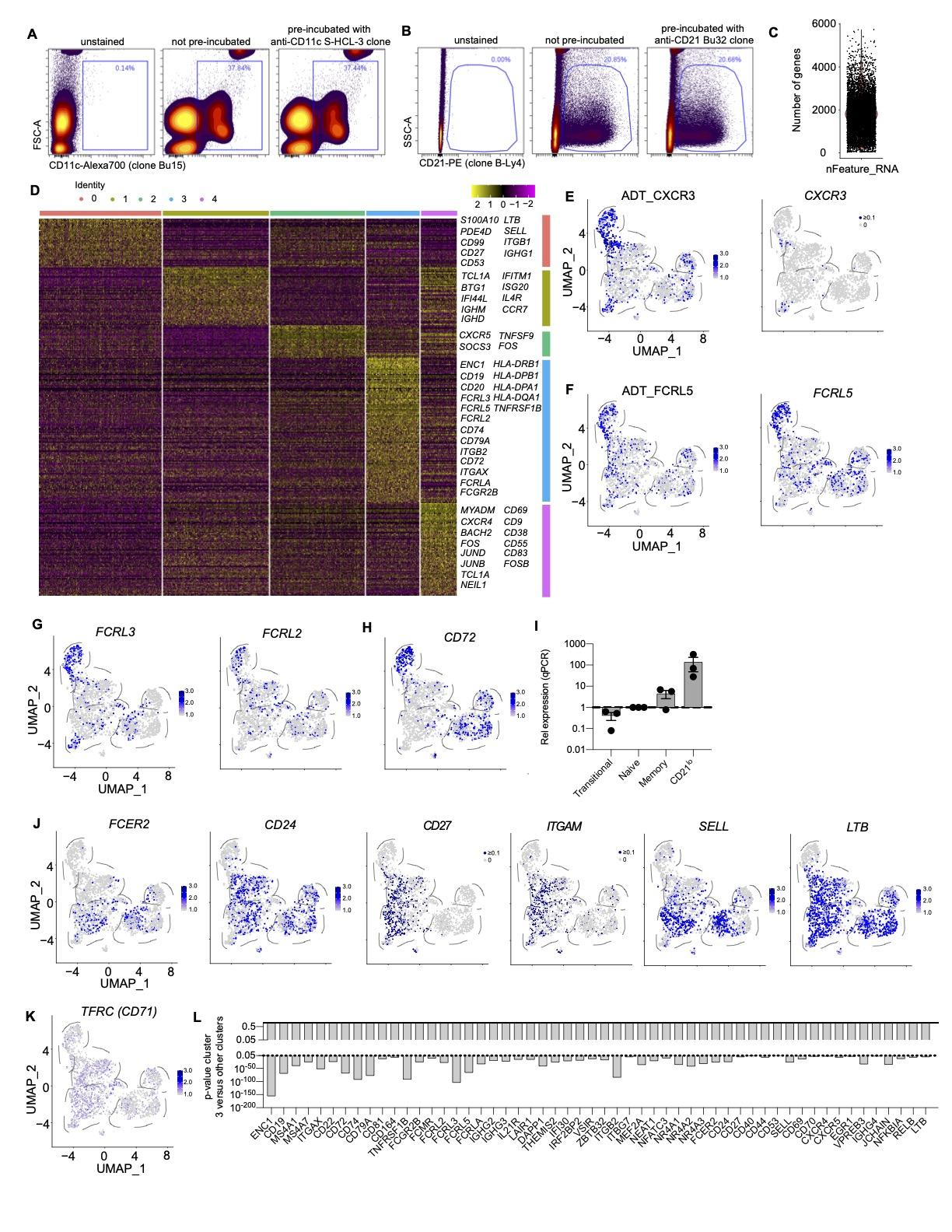
**

**Figure S5. Proteotranscriptomic investigation of CD21^lo^ B cells from a patient with inherited human T-bet deficiency. (A)** In a trial experiment, PBMCs from a healthy donor were labeled with or without oligonucleotide-conjugated anti-CD11c (clone S-HCL-3), and were subjected to surface staining with anti-CD11c-Alexa700 Ab (Clone Bu15). Surface expression of CD11c and FSC-A were plotted. **(B)** In a trial experiment, PBMCs from a healthy donor were labeled with or without oligonucleotide-conjugated anti-CD21 (clone B-Ly4), and were subjected to surface staining with anti-CD21-PE Ab (Clone B-Ly4). Surface expression of CD21 and SSC-A were plotted. **(C)** PBMCs from age-matched healthy donors (Age-CTL), IFN-γR1-deficient (IFN-γR1 ^-/-^) and T-bet-deficient (M/M) patients were labeled with hashtag Abs and oligonucleotide-conjugated anti-CD11c, anti-CD21, anti-CD95, anti-CXCR3, anti-FcRL5 Abs. CD3^-^CD56^-^CD20^+^CD21^lo^ B cells were isolated by FACS and subjected to CITE-seq. Numbers of genes expressed by each single cell in this study were plotted. **(D)** CD21^lo^ B cells from two age-matched controls, an IFN-γR1 ^-/-^ patient, and P (M/M), as in (C), were subjected to dimensionality reduction by UMAP based on their transcriptome. Heat map showing the most significantly upregulated genes of clusters 0, 1, 2, 3, and 4 of CD21^lo^ B cells from these four samples. **(E)** Cells expressing surface CXCR3 protein and *CXCR3* mRNA were highlighted in UMAP plots. **(F)** Cells expressing surface FCRL5 protein and *FCRL5* mRNA were highlighted in UMAP plots. **(G)** Cells expressing *FCRL2* and *FCRL3* mRNA were highlighted in UMAP plots. **(H)** Cells expressing *CD72* mRNA were highlighted in UMAP plots. **(I)** Naïve, memory, transitional, or CD21^lo^ B cells isolated with FACS were subjected to quantitative RT-PCR analysis of the expression of *ENC1*. *ENC1* expression was shown. **(J)** Cells expressing *FCER2*, *CD24*, *CD27*, *ITGAM*, *SELL*, and *LTB* were highlighted in UMAP plots. **(K)** Cells expressing *TFRC* (*CD71*) were highlighted in UMAP plots. **(L)** P-value of comparison between cluster 3 and other clusters combined of indicated genes.

**
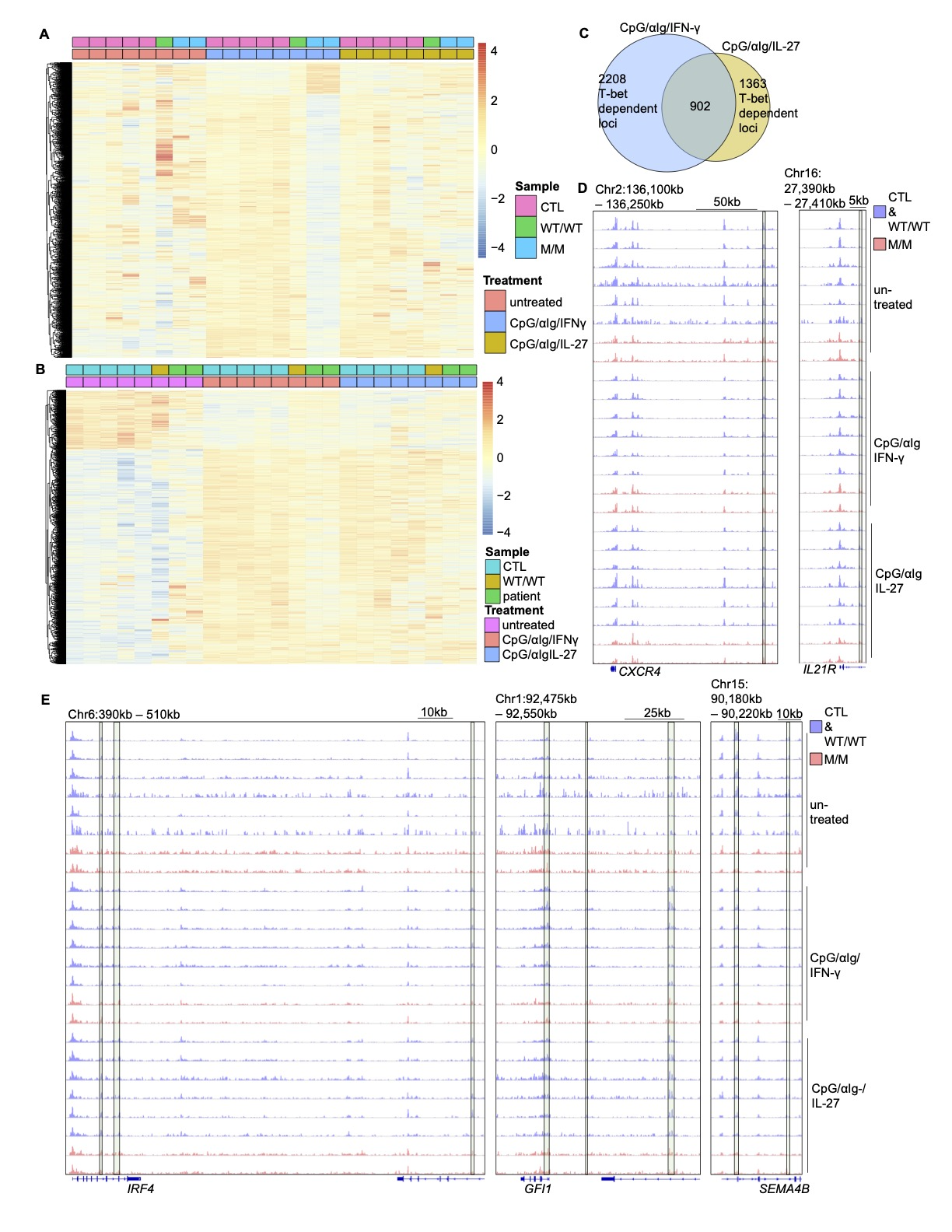
**

**Figure S6. Chromatin accessibilities of B cells are altered in inherited T-bet deficiency.** (**A**) CD3^-^CD56^-^CD20^+^CD27^-^CD10^-^IgG^-^ naive B cells were isolated from the PBMCs of controls (CTL) and P (M/M) by FACS. They were left untreated or stimulated with a BCR agonist, anti-Ig Fab (αIg), or a TLR9 agonist, CpG, in the presence of IFN-γ and IL-27. All loci with differentially regulated chromatin accessibilities are shown. (**B**) Heat map showing the chromatin accessibilities of all loci differentially regulated in response to IFN-γ or IL-27 in controls (CTL), with no significant difference in accessibility in the B cells from P (M/M). (**C**) The numbers of T-bet-dependent loci, the chromatin accessibilities of which were differentially regulated in control cells, but remained not significantly different, in response to αIg+CpG+IFN-γ or αIg+CpG+IL-27. (**D**) Chromatin accessibility of the *CXCR4* and *IL21R* loci, which were differentially regulated in response to αIg+CpG+IFN-γ and αIg+CpG+IL-27 in control B cells but not in T-bet-deficient B cells. (**E**) Chromatin accessibility of the *IRF4*, *GFI1*, and *SEMA4B* loci, which were differentially regulated in response to αIg+CpG+IFN-γ and αIg+CpG+IL-27 in control B cells but not in T-bet-deficient B cells.

|  | Age | | |  |
| --- | --- | --- | --- | --- |
| Parameters | 6 months | 2 years | 3 years | Threshold |
| IgG anti-tetanus toxoid | ND | ND | **0.20** | >0.1 |
| IgG anti-diphtheria toxoid | ND | ND | **0.33** | >0.1 |
| IgG anti-*Haemophilus* b | ND | ND | **0.205** | >0.11 |
| Pneumococcal IgG antibodies | ND | ND | **27.1 µg/mL** | >0.25 µg/mL |

**Table S1. Vaccine-specific antibody responses in T-bet deficiency.** Plasma samples prepared when the patient was 6-month-, 2-year-, and 3-year-old were measured for the levels of antigen-specific antibodies. Units are IU/mL if not otherwise specified. ND not detectable.
